# Mind-Personality Relations from Childhood to Early Adulthood

**DOI:** 10.3390/jintelligence6040051

**Published:** 2018-12-06

**Authors:** Andreas Demetriou, George Spanoudis, Mislav Stjepan Žebec, Maria Andreou, Hudson Golino, Smaragda Kazi

**Affiliations:** 1 Department of Psychology, University of Nicosia, Nicosia 1700, Cyprus; 2 Department of Psychology, University of Cyprus, Nicosia 1678, Cyprus; spanoud@ucy.ac.cy (G.S.); maria.polydorou@cytanet.com.cy (M.A.); 3 Department of Psychology, Croatian Studies University of Zagreb, 10000 Zagreb, Croatia; mzebec@hrstud.hr; 4 Department of Psychology, University of Virginia, Charlottesville, VA 22903, USA; hfg9s@virginia.edu; 5 Department of Psychology, Panteion University of Social Sciences, 17671 Athens, Greece; smakazi@otenet.gr

**Keywords:** personality, intelligence, development, cognition

## Abstract

We present three studies which investigated the relations between cognition and personality from 7 to 20 years of age. All three studies showed that general cognitive ability and the general factor of personality are significantly related throughout this age span. This relation was expressed in several ways across studies. The first investigated developmental relations between three reasoning domains (inductive, deductive, and scientific) and Eysenck’s four personality dimensions in a longitudinal-sequential design where 260 participants received the cognitive tests three times, and the personality test two times, covering the span from 9 to 16 years. It was found that initial social likeability significantly shapes developmental momentum in cognition and vice versa, especially in the 9- to 11-year period. The second study involved 438 participants from 7 to 17 years, tested twice on attention control, working memory, reasoning in different domains, and once by a Big Five Factors inventory. Extending the findings of the first, this study showed that progression in reasoning is affected negatively by conscientiousness and positively by openness, on top of attention control and working memory influences. The third study tested the relations between reasoning in several domains, the ability to evaluate one’s own cognitive performance, self-representation about the reasoning, the Big Five, and several aspects of emotional intelligence, from 9 to 20 years of age (N = 247). Network, hierarchical network, and structural equation modeling showed that cognition and personality are mediated by the ability of self-knowing. Emotional intelligence was not an autonomous dimension. All dimensions except emotional intelligence influenced academic performance. A developmental model for mind-personality relations is proposed.

## 1. Introduction

Mental processes and motivational and personality dispositions interact to guide understanding and action in the world. Wechsler argued that “general intelligence cannot be equated with intellectual ability but must be regarded as a manifestation of the personality as a whole” [1] (p. 83). Allport [2] (p. 48) suggested that personality is “the dynamic organization within the individual of those psychophysical systems that determine his unique adjustment to his environment”. In Cattell’s [3] model intelligence and personality occupy equal standing. However, there is no generally accepted model specifying when and how cognitive and personality processes interact. Recognizing this lack, Jensen [4] concluded “The g factor”, his magnum opus on general intelligence, urging researchers to study the intelligence-personality nexus. 

The studies presented here explore this nexus, tracing its changes in development. It is well known that, on the one hand, individuals at different ages understand the world differently despite possible similarities in personality [5]; on the other hand, early differences in personality may channel individuals to relate differently with the world, despite their intellectual similarities, eventually differentiating their developmental opportunities [6,7]. For instance, reflective individuals may control motivational and personality dispositions, achieving better adaptation in their environment than less reflective individuals. No satisfactory model exists for these interactions. This paper presents three studies designed to highlight the relations between cognition and personality from several points of view. Specifically, all three studies examined (i) if cognition and personality are systematically related; (ii) how different component process in each interact with components in the other; (iii) if there is a central mediator underlying these interactions; and (iv) disentangle their relative influence on real life outcomes such as academic achievement. Below we review the literature related to the organization of intelligence and personality, their development, and their possible interactions in development. This introduction concludes with predictions suggested by this research. 

### 1.1. The Organization of Cognition and Personality: Commonalities and Differences

In recent years, there has been significant progress in the study of the organization of intelligence and personality and their possible common ground (e.g., [8,9,10]). There is consensus that both are hierarchically organized. Specifically, intelligence is a three-level hierarchical structure involving three types of systems: (i) several broad domains of ability, such as spatial, verbal, and quantitative reasoning; (ii) domain-general systems of inferential and representational processes, such as *fluid intelligence* (Gf; basically inductive reasoning allowing processing of similarities and relations at increasing levels of abstraction) and *crystallized intelligence* (Gc; knowledge and skill systems crystallizing learning and experience that may be activated for understanding and problem solving); (ii) a general factor (g) mainly associated with processing efficiency, attention control, mental flexibility, and working memory [4,11,12,13]. Cognizance was recently added as a major background process of g. This includes a suit of processes including self-monitoring of mental and behavioral processes, representation and awareness of them, reflection on and intentional regulation of them, and metarepresentation, which generates new mental and behavioral constructs out of the modification and integration of available constructs [5,14,15,16]. 

*Personality.* In personality, a structurally equivalent hierarchical model is gaining popularity. This model specifies several dimensions standing for specific dispositions to relate with the world. According to the Big Five Factors model, which dominates the field, these dimensions are: *extraversion* (E, enjoying being with others and actively seeking social company and activity); *agreeableness* (A, orientation to others, trusting them and be warm with and make good to them); *conscientiousness* (C, goal-minded, focused, careful, organized, determined, and planful; *neuroticism* (N, individuals high in neuroticism are disturbed by variations in the environment so that they are nervous, anxious, and moody); *openness to experience/intellect* (O, being curious, inventive, original, and imaginative with wide interests and trying new experiences). The Big Five Model integrated constructs from earlier models of personality. It shares two of the Big Five with Eysenck’s [17] theory (E and N) and overlaps with the third, psychoticism (P), which combines traits of A and C [18,19,20]. It also integrated openness from Cattell’s [3] model, standing for the projection of mental ability in personality. 

Evidence shows that three of the Big Five Factors relate to one and the rest to another second-order factor: C, N (emotional stability), and A relate to the general trait of stability, the α-factor, underlying efficiency in organizing one’s own life, dealing with pressure, and making oneself acceptable; the other, the beta factor (β-factor), relates with O and E, and stands for plasticity in one’s relation with the world. In turn, these two factors relate to a third-order general factor, the general factor of personality (GFP). Broadly speaking, these factors appear related to the higher-order factors in the hierarchical model of intelligence outlined above. Stability associates with crystallized intelligence, including dispositions and skills underlying interactions with the social world; plasticity expresses fluid intelligence in personality. “The GFP is analogous to g and predicts social efficiency in the way g predicts cognitive efficiency” [21] (p. 564). GFP, like g, relates to actual life indicators, such as performance at school and work (e.g., [22]). 

Despite their possible similarities in overall structure, there is a large uncharted territory between intelligence and personality. For instance, we do not yet know if there is a super-G, over g and the GFP, capturing processes and functions integrating mental and personality functioning. This would stand for the whole person, gP, and it would represent an individual’s mental and behavioural uniqueness. For instance, one might claim that g, general mental power, is projected into one’s self-concept [23] as general self-efficacy [24], confidence in self-evaluations of performance across domains [25], and even Freud’s ego and ego strength [26]. That is, g, standing for outcomes of the individual’s cognitive activity as measured by the researcher, and the GFP, standing for one’s own evaluation and representation of these outcomes share a common ground, success in problem solving and interactions and evaluations of it—carried out by oneself and others and mutually broadcasted. 

It is interesting that the general factor is disputed in both fields. In intelligence, scholars argue that g does not really exist; in their view, g is a statistical artefact reflecting the interaction between processes activated according to the requirements of the current task rather than any specific mental process [27,28]. Along the same line, in personality, scholars claim that GFP may be a technical artefact, additionally reflecting a generally present likeability tendency reflected in all Big Five Factors [29]. Currently, the evidence about the commonalities between intelligence and personality is inconsistent. On the one hand, research on the genetic (e.g., [30]) and the brain basis [31,32] of intelligence and personality suggests that specific genetic profiles channel brain formation and functioning imposing common constraints on mental and personality functioning. For instance, specific genes channel the operation of neurotransmitters, such as dopamine and serotonin, which relate to psychological characteristics common to both intelligence and personality, such as novelty seeking and emotional reaction to stimulation [33,34]. On the other hand, however, there is evidence that the genetic correlation between the general factors of intelligence and personality is 0 [35]. Obviously, systematically establishing or falsifying the possible relation between g and the GFP is important for several lines of ongoing research. All three studies presented here shed light on this question. 

Specifically, we will examine if there are common background processes operating in both personality and intelligence. We will focus on two distinct but related processes: executive control and cognizance. Executive control, the ability to focus on stimuli, plan action, and flexibly change focus as needed, is a central component of Gf in current models of intelligence [36]. Cognizance is an active translation agent that transforms executive control experiences into regulation schemes that may be used in managing inferential processes and vice versa [15,16,37]. In personality, these mechanisms may be involved in two different dimensions. As mechanisms of regulation and planning, they may underlie the self-discipline and stability involved in C; as mechanisms of flexibility, they may be instrumental for sustaining plasticity in dealing with others (E) or with novelty (O) [7]. We know of no research that explored these questions. It is noted, however, that all personality theories, including the Big Five Factor model, are largely based on self-rating inventories. Therefore, personality research and theory take for granted that there is a self-monitoring and self-recording agent that is minimally accurate to describe dispositional and behavioural actualities: responses to personality inventories are generated by this agent. Indeed, there is strong evidence that the GFP is highly self-representational, reflecting a person’s self-concept and self-worth rather than actual cognitive ability: the relation between GFP and self-esteem is very high (67% of the variance) [38]. Also, self-represented cognitive g accounts for much more of the variance of all Big Five (between 18–25%) but N (1%) than Gf (between 1–3%) [39]. We assume that these self-concepts are products of the operation of cognizance, which varies in accuracy and scope with development [5,15,38,39]. Therefore, it is important to specify how cognizance relates to the various intelligence and personality factors and mediates between them at successive developmental phases. 

### 1.2. Development of Cognition and Personality

*Cognitive development*. There is general agreement that intelligence develops over a series of levels from birth to early adulthood. Macroscopically, theories of cognitive development agree that major transitions occur around the age of 2, 7, and 11 years, when thought changes in abstraction, cohesion, and reasoning power. These changes were associated with increasing processing and representational efficiency as indicated by functions such as processing speed, attention control, and working memory [40,41,42]. Integrating over these theories and a long series of empirical studies, we postulated that cognitive development occurs in four major developmental cycles, with two phases in each. New representations emerge in the first phase of each cycle and their integration dominates in the second. Transitions across cycles is driven by cognizance, i.e., increasing self-awareness of mental representations and processes, reflection, metarepresentation engendering new representations, and ensuing self-regulation [5,16]. 

In succession, the four cycles operate with episodic representations from birth to two years, realistic mental representations from 2 to 6 years, generic rules organizing representations into conceptual/action systems from 6 to 11 years, and overarching principles integrating rules into systems where truth and multiple relations can be evaluated from 11 to 18 years. Changes within cycles occur at about their middle, at 4, 8, and 14 years, when representations become explicitly cognized so that their relations can be worked out, gradually resulting into representations of the next cycle. Therefore, these cycles are distinguished from each other by the type of representation dominating in each (i.e., episodic schemes, mentations, rules, and principles) and the relations connecting representations (i.e., spatially and time-based associations, representational mappings, inferential links, truth- or validity-based inferential constraints) [5,16]. 

Cognizance is central in this developmental system. It defines the subjective aspect of mental functioning, raising it from simple computation to representation where information and mental functioning is subjectively meaningful. Thus, cognizance is a major source of transitions across cycles. The self-representation system gradually builds pointers to different combinations of (i) problem solving skills and processes; (ii) dispositions to go on with a particular pattern of activity or abandon it; and (iii) feedback received about successes and failures and the ensuing feelings of satisfaction and dissatisfaction. These pointers are used by the person for self-regulation and self-representation, enabling him or her to choose appropriate action patterns among alternatives. Thus, both action patterns and self-representations come out as packages involving combinations of abilities, dispositions, styles, and interests. Cognizance is cycle-specific, exerted through the representational processes dominating in each cycle: it is based on perception- and iconic-like representations in the representational cycle; rule-based inferential processes in the rule-based cycle; and abstract semantic processes in the principle-based cycle. It becomes increasingly accurate along the cycles resulting in an increasingly refined understanding of the functioning of the mind and increasingly accurate self-representations, specifying personal strengths and weakness [5,14,15,16]. 

*Personality development.* Precursors of adult personality dimensions are established very early in life. Temperament, which reflects differences between children in their reactivity to external stimuli and their ability for self-regulation are present since infancy. For instance, the tendency to independently explore the environment which predates openness and the tendency to become distressed by variations in the environment which predates neuroticism are present from the first months of life [7]. Thus, the Big Five Factors are discernible from early childhood; however, their reliability and stability increase with age [43,44]. Overall, conscientiousness, irritability, and positive activity are present and relatively stable since early childhood; extraversion and neuroticism stabilize after the age of eight years; openness may not be a meaningful dimension of personality prior to adolescence [45,46]. 

### 1.3. Interactions between Intelligence and Personality

Some relations between cognitive processes and personality dispositions are well established in the literature. For instance, the positive relation between fluid intelligence and openness is well established [8]. In development, Ziegler [10] showed longitudinally that openness positively influences the development of fluid intelligence from adolescence to early adulthood. There is evidence that open individuals ‘see’ more possibilities in the input and they flexibly combine information from the two eyes in a creative fashion, especially under a positive mood [47]. Along this line, personality differences influence selective attention to stimuli. McIntyre and Graziano [48] showed that individuals oriented to other persons tend to selectively attend to social stimuli; individuals oriented to inanimate things tend to selectively attend to objects. Finally, there seems to be a negative relation between conscientiousness and intelligence, implying that intelligent individuals are less organized and rule-abiding. Noticeably, however, both conscientiousness and intelligence are positively related to various life outcomes, such as school success, implying that organizing behaviour in the pursuit of long-term goals may compensate for possible limitations in intelligence [9,39]. Overall, in developmental models of intelligence-personality relations the direction of causality is tilted in favour of personality. Originating from differential rather than developmental theory, the models assume that some personality dispositions allow better “investment” of available cognitive ability in activities conducive to learning thereby increasing intelligence or life achievements [49,50,51]. Specifically, several investment traits, such as curiosity, need for cognition, novelty seeking, openness to experience, and mindfulness, when high, drive the person to engage in cognitive activities which cause cognitive enhancement.

Based on the discussion above, the studies presented here are relevant to the following predictions. First, psychological research accesses cognition and personality at different levels of functioning. Cognitive measures reflect performance as measured by the researcher. Personality measures reflect self-representations of one’s own behavioral tendencies or behavior. Therefore, cognition-personality relations may be masked by differences in variation of the two levels involved (actual cognitive tasks vs. self-representations and personal experiences) and differences in measurement accuracy of those involved (the researcher vs. the participant). Hence, correlations at the task and domain level may be low; however, relations between latent constructs capturing general cognitive and personality dimensions would be systematically present, because latent constructs capture mental and behavioral constraints exceeding local task- or experience-related variation. 

Second, relations between mental processes and personality dispositions would be bidirectional and would strengthen with age, reflecting the increasing consolidation of personally adaptive patterns of understanding and behavior. These relations would reflect the nature of self-understanding and mental complexity possible at the developmental cycle concerned. For instance, cognition-personality relations would be closer in adolescence than in childhood because self-monitoring and self-regulation are more accurate and efficient in the cycle of principle-based thought as compared to the cycle of rule-based thought. Additionally, in periods of extensive cognitive restructuring cognition-personality relations may strengthen to reflect the transfer of cognitive changes to overall self-representation, self-evaluation, and self-management. 

Third, given the self-representational nature of personality evaluation, cognizance would emerge as the main link between cognition and personality. This may emerge in several forms. When directly measured, cognizance would emerge as the main mediational construct bridging cognition with personality. When represented by a proxy, such as Eysenck’s social likeability or cognitive self-concept as such, which reflect personal value-laden self-representations, relations between the proxy and cognition would be stronger than between cognition and other personal dispositions, such as neuroticism. These differential relations would reflect influences of cognitive change on the fine-tuning of one’s self-representation and self-worth vis-à-vis the world. 

Fourth, some cognitive processes are related with personality processes more than with others and vice versa. Specifically, executive mental processes, such as attention control, would relate with personality processes reflecting plasticity, such as openness; inferential processes would relate with stability processes, such as conscientiousness and agreeableness, reflecting the fact that these personality dispositions directly use these processes in developing self-organization and social interactive aspects of behavior. 

Finally, fifth, actual life outcomes, such as academic achievement, would equally relate to all three aspects of dealing with the world, cognitive, personality, and motivational, because all three of them are involved in sustaining long-term behavioral and socially valued goals [9,39]. 

## 2. Study 1: Developmental Changes in Personality-Intelligence Relations

The present study was part of a longitudinal study of cognitive development covering the age span from 9 to 16 years of age (see [52]). The study focused on the development of inductive (fluid) reasoning, deductive syllogistic reasoning, and scientific reasoning underlying the ability to test hypotheses by specifically designed experiments. Participants were tested three times separated by one-year intervals by a specifically designed Raven-like Matrices test, a test of syllogistic reasoning addressed to various logical schemes, and a test of scientific reasoning requiring isolation of variables in designing experiments at several levels of complexity [53]. At the first two testing waves participants were also tested by the Eysenck Personality Questionnaire (EPQ) [54]. Therefore, this study allows longitudinal testing of how cognitive changes relate with possible personality changes in the transition from childhood to adolescence. 

Eysenck’s theory of personality specified four factors: extraversion (E), neuroticism (N), psychoticism (P), and social likeability (L). We noted above that two of these factors, E and N, are by and large the same with the corresponding factors in the Big Five Factor model. There is less agreement about the rest two. Specifically, P is characterized by aggression, assertiveness, egocentric and manipulative behaviors, orientation to achievement, dogmatism, and tough-mindedness. Notably, Eysenck [19] himself and Costa and McCrae [20], the proponents of the Big Five Factor model, agree that P involves a combination of traits of A and C. Empirical evidence did show that P involves C and Impulsive Sensation Seeking, indicating lack of planning and a tendency to act without thinking [55]. Individuals high in L tend to give positive characteristics to themselves in their relations with others: i.e., that they are considerate, good-mannered, and faithful to others, enjoying being with and co-operating with them, follow the rules, and recognize their mistakes. This scale correlates with Impulsive Sensation Seeking [55]. 

Customizing the general predictions stated above to the present study allows testing three specific predictions. First, according to the first prediction about general factors, systematic relations between g and the GFP are expected. Second, according to the second prediction about the direction of cognition-personality interactions, stronger cognition-personality relations are expected in the earlier part of the age period examined here to signify the transition from rule- to principle-based thought from childhood to adolescence. Third, according to prediction about the role of cognizance, a privileged relation of L to cognitive change relative to the other personality dimensions is expected to reflect adjustments in self-evaluation and self-representation associated with this transition. Finally, according to prediction about varying relations between different mental and personality processes, some dimensions, such as P may be negatively linked to cognitive change to reflect relative caution in cognitive engagement which may deprive individuals of possible learning opportunities. 

### 2.1. Method

#### 2.1.1. Participants

A total of 260 participants were examined longitudinally, covering the age span from 9 to 16 years. Specifically, this total included 44 4th grade primary-school children (mean age 9.6 years old at first testing; 31 males), and 46 (mean age 12.6 years at first testing; 21 males), 92 (mean age 13.6 years at first testing; 46 males), and 78 (mean age 14.6 years at first testing, 31 males) 1st, 2nd, and 3rd grade secondary school students, respectively. All cohorts were tested three times by the cognitive battery and two times by the EPQ (in March or April) separated by one-year intervals. Participants lived in Thessalonki, the second largest city in Greece, and came from middle or high SES families. 

#### 2.1.2. Task Batteries

*The fluid reasoning battery* included 12 Raven-like matrices specifically designed to address the following four levels of complexity: (i) integration of two attributes varying in the same direction (e.g., geometric figures of increasing size and with a background mark increasing in the same direction); (ii) integration of intersecting elements, such as size and shape, marked by elements such as dots at a particular position, without transformations; (iii) dissociation of relevant from irrelevant elements or simple transformations such as the rotation of a line across matrices; and (iv) integration of multiple properties varying according to several rules (e.g., change in shape, size and position of some attribute). 

*The deductive reasoning battery* included 20 items addressed to different types of logical relations scaling in four levels: (i) Modus ponens (implication) and disjunctive reasoning; (ii) modus tollens and falsification of disjunctive propositions; (iii) understanding of arguments of falsification and grasp of non-decidability of arguments; (iv) explicit grasp of the rules of implicative reasoning and full mastery of fallacies (affirmation of the consequent and denial of the antecedent). 

*The scientific reasoning battery* included 16 items addressed to the following four levels: (i) identification of confounding variables and interpretation of the results of simple experiments (matching variables with their effects); (ii) systematic isolation of variables of explicit hypotheses; (iii) design experiments verifying a hypothesis and interpretation of evidence suggesting various causal relations (necessary and non-sufficient; neither necessary nor sufficient; incompatible); (iv) design experiments (i) to falsify a hypothesis; (ii) verify each of the above causal relations, and model construction. 

The full version of the *Eysenck Personality Questionnaire* (EPQ) was used. EPQ involves 80 items addressed, 20 for each four personality dimensions specified in Eysenck’s theory [17]. 

#### 2.1.3. Scoring

All items in the cognitive batteries were scored on a pass-fail basis. To pass a matrix, two empty cells (the second and third cell of the second and third raw, respectively) would have to be filled in by choosing the proper options among eight alternatives. To pass a syllogism, the logically proper answer would have to be selected among three alternatives. To pass a scientific reasoning item answers would have to indicate task-relevant control of variables, hypothesis formation, or data interpretation. 

A level score was given to each participant for each battery; this is the highest level at which a person solved two thirds or more of the tasks at this level. The level structure of each battery was validated and fine-tuned by means of discrimination level analysis. This method was developed by Shayer to score individual performance on batteries involving developmentally scaled tasks [56]. This method showed that the four-level sequence proved very consistent for each battery. In concern to the developmental cycles summarized in the introduction, the first two levels of the inductive and deductive reasoning batteries addressed early and late rule-based thought and the last two addressed principle-based thought. Level I of the scientific reasoning battery addressed late rule-based thought; level II addressed early principle-based thought; levels III and IV addressed late-principle-based thought. Thus, a level score, ranging from 0 (not satisfying ascription to the lower level of a scale) to 4 was ascribed to each person for each battery at each testing wave. These scores were used in the various analyses to be presented below. All three batteries were very reliable (Cronbach’s alpha always >0.8; mean inter-rater agreement for the scientific reasoning battery was 85.2%, sd = 9.7). Within battery correlations of level scores across waves were high (0.45–0.67); correlations across batteries were lower, but still high (0.29–0.56). 

Responses to the items in the EPQ were identified as yes/no (applies vs. does not apply to me) and the participant’s score on each dimension was the sum of the items judged to apply. Cronbach’s alpha was marginally satisfactory if estimated at each testing wave (0.51 and 0.47 for first and second testing, respectively); however, it was high if the reliability of the two testing waves was estimated together as used in the structural equation models tested (0.75). Self-correlations across waves were very high: 0.72–0.85; N and P correlated positively with each other within, and across, waves (0.18–0.22); E correlated positively with P (0.11–0.13) and negatively with N (−0.13 to −0.15); L correlated highly and negatively with P and N (−0.39 to −0.49) and negatively but low with E (−0.05 to −0.09). Correlations of three personality dimensions (E, P, and N) with the three cognitive scales were low to moderate (0.00–0.26); the correlations of L with the three cognitive dimensions were much higher, but always negative (−0.22 to −0.45), suggesting a systematic developmental relation (see correlations in Table S1, in the Supplementary Materials).

### 2.2. Results

**Figure 1:**
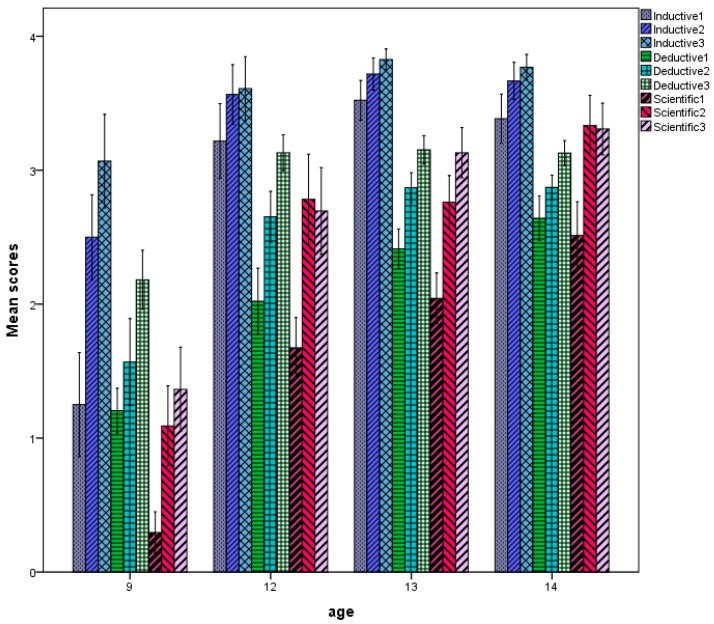
Performance on the three cognitive batteries across age and testing waves.

The pattern of performance on the cognitive tests is shown in Figure 1. The effect of age, F (3, 252) = 120.415, *p* < 0.0001, partial η^2^ = 0.59, was highly significant and very powerful, reflecting large improvement of performance across age groups. The effect of testing wave was also significant, F (2, 251) = 238.813, *p* < 0.0001, partial η^2^ = 0.66, reflecting large improvement across testing waves. The effect of cognitive domain was also highly significant, F (3, 251) = 259.668, *p* < 0.0001, partial η^2^ = 0.67, reflecting variation of performance across domains: performance on inductive reasoning was higher than on deductive reasoning and this was higher than scientific reasoning. The interactions between age and wave F (6, 504) = 7.732, *p* < 0.0001, partial η^2^ = 0.08, and wave and domain, F (4, 249) = 7.756, *p* < 0.0001, partial η^2^ = 0.11, were also significant, indicating two trends: first, the magnitude of improvement across waves varied with age; it was much larger at lower ages, indicating transition from rule- to principle-based thought and approaching ceiling at older ages; the second change was almost linear across domains in inductive and deductive reasoning but larger from first to second than from second to third in scientific reasoning. Noticeably, no effect of gender ever reached significance.

**Figure 2:**
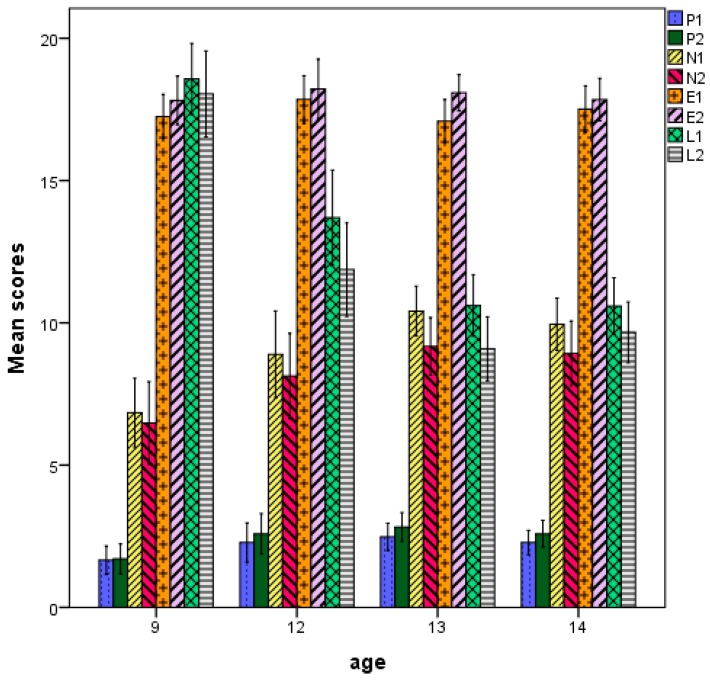
Personality scores across age, testing wave, and personality dimensions.

There were similarities and differences in the pattern of relations between age, testing wave, and personality dimensions. Specifically, the effect of age, F (3, 256) = 9.307, *p* < 0.0001, partial η^2^ = 0.10; wave, F (1, 256) = 15.390, *p* < 0.0001, partial η^2^ = 0.06, and dimension was significant, F (3, 254) = 1811.559, *p* < 0.0001, partial η^2^ = 0.96. Additionally, the age by dimension, F (9, 768) = 8.264, *p* < 0.0001, partial η^2^ = 0.09, and the waves by dimension interaction was highly significant, F (3, 256) = 28.287, *p* < 0.0001, partial η^2^ = 0.26, indicating that the relation with age differed across dimensions. Figure 2 illustrates these effects: P and N increased across the first three age groups and decreased at the last age group; scores in E increased from 9 to 11 years, decreased from 11 to 13, and then increased again; scores in L decreased across the first three age groups and stabilized there. Across waves, P and E increased; N and L decreased. Thus, in line with our first prediction, changes in personality were larger earlier in age, largely reflecting patterns of cognitive change. 

### 2.3. Cognition-Personality Relations with Development

Three approaches were adopted to specify the relations between changes in mental and personality processes. First, a series of confirmatory factor analyses examined the robustness of cognitive and personality factors and if these factors are related. A first pair of models focused on performance at first testing. In this model, performance on inductive, deductive, and scientific reasoning at first testing were related to one factor standing for Gf and the scores on the four personality factors at this testing were related to another factor standing for the GFP. In the first of the two models the correlation between these two factors was constrained to 0 (χ^2^ (14) = 115.79, CFI = 0.76, *p* < 0.001, RMSEA = 0.168, AIC = 87.79). In the second model, the correlation of the two factors was left free to be estimated, (χ^2^ (13) = 38.54, CFI = 0.94, *p* < 0.001, RMSEA = 0.087, AIC = 12.54). The fit difference between the two models was significant, Δχ^2^ (1) = 77.25, *p* < 0.001, reflecting the fact that the two factors were significantly correlated (0.59) (see Study 1, Model 1, in Supplementary Materials presenting model codes and complete solutions). The pattern of results obtained from the models applied to the second testing wave were practically identical with those of the first testing. It is notable, however, that the same pattern emerged when this approach was applied on the performance attained at all testing waves. Specifically, in this model, a Gf factor was created for each of the three testing waves associated with performance on inductive, deductive, and scientific reasoning at the respective wave. In the same fashion, a GFP of personality was created for each of the first two testing waves, associated with the scores on each of the four personality factors at the respective wave. The three wave-specific Gf factors were regressed on one second-order factor and the two wave-specific personality factors were regressed to another second-order factor. In a sense, then, these two factors stand for diachronic cognitive ability and personality operating regardless of developmental changes in each of them, in the time-window covered by this study. Therefore, it is highly interesting and relevant to the analyses to follow to specify how these factors are related. In the fashion above, in the first model, the correlation between the two diachronic factors was constrained to be 0, (χ^2^ (105) = 293.64, CFI = 0.92, *p* < 0.001, RMSEA = 0.084, AIC = 83.64). In the second model, this correlation was left free to be estimated, (χ^2^ (104) = 213.72, CFI = 0.95, *p* < 0.001, RMSEA = 0.064, AIC = 5.72). The difference between the fit of the two models was significant, Δχ^2^ (1) = 79.92, *p* < 0.001, reflecting the fact that the two factors were highly correlated (0.76). Notably, when this second model was estimated after partialling out the effect of age from each score-factor relation involved, the model still held well, (χ^2^ (104) = 200.40, CFI = 0.96, *p* < 0.001, RMSEA = 0.060, AIC = −7.60); the correlation between the two factors dropped, but it was still significant and high, (0.48). Therefore, in line with the first prediction about general factors, cognition and personality are systematically related in the period from 9 to 16 years and this interaction is developmentally sensitive to developmental influences. The models below will specify the sources of these interactions. 

**Figure 3:**
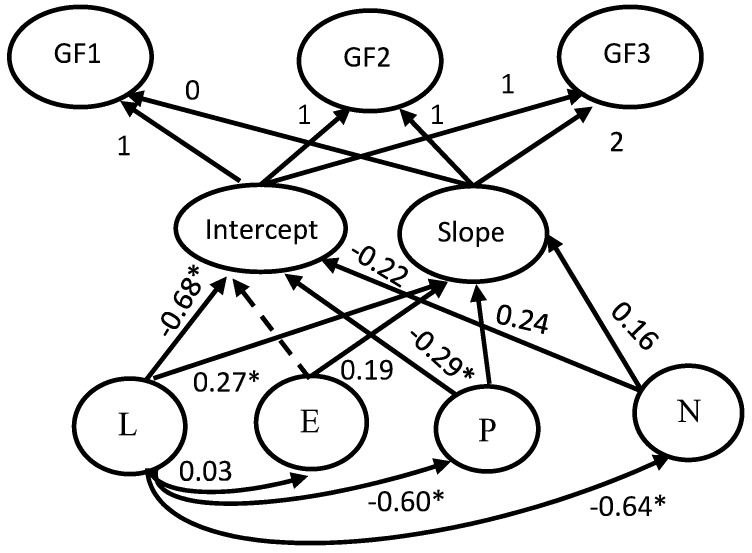
The growth model of Gf with personality effect on Gf intercept and slope. Note: See the full model in Supplementary Materials (Study 1, Model 2) presenting model codes and complete solutions.

Second, a growth model examined the possible influence of personality on the form of cognitive development in the three years covered by this longitudinal study. This model is shown in Figure 3. It may be seen that all three cognitive scores of each testing wave were related to a wave-specific Gf factor. The relation between these three factors and the intercept was constrained to unity to capture the initial mean of the growth function. To specify the degree of change across the three testing waves, the relation between the three wave-specific factors and slope was constrained to 0, 1, and 2, respectively. To specify the possible distinct influence of personality on the intercept and slope, P, N, and E were regressed on L, thereby raising L to a background factor standing for the GFP (see Figure 3). The intercept and slope of Gf were regressed on L and the *residuals* of each of the other three personality factors. This manipulation allows to distinctly specify the effects of L and each of the other three personality factors on the two Gf growth parameters, after removing any likeability possibly involved in them. The fit of this model was very good, Satorra-Bentler χ^2^ (12) = 16.27, CFI = 0.965, *p* = 0.180, RMSEA = 0.071. It may be seen that the intercept was negatively and highly related to the L factor (−0.68) and moderately but significantly to the P (−0.29) and the N (−0.21). These effects suggest that initial high scores in L but also in factors reflecting low emotional stability are associated with comparatively lower cognitive performance. 

However, the relation of personality factors with slope was significant and positive (0.27, 0.17, 0.24 for L, E, and P, respectively). This implies that initial high scores in these factors were associated with larger cognitive change. 

Third, to further specify these relations, latent transition analysis (LTA) was employed [57]. LTA specifies how individuals move across categories in a period of interest and the factors possibly affecting this movement. Here the first two waves were involved. Two categories were specified, one for cognitive performance at the first wave and one for cognitive performance at the second wave. The level score of each of the three cognitive abilities at each testing wave were used. There were two classes of participants in each category: those changing into a higher level and those staying on or regressing from their initial level. The mean score on each of the four personality factors were involved as covariates to examine how personality factors affect transition to a higher level across the first two testing. Practically, we regressed transition to a higher level across the two waves on these personality scores to examine how they influence the likelihood of change. 

**Figure 4:**
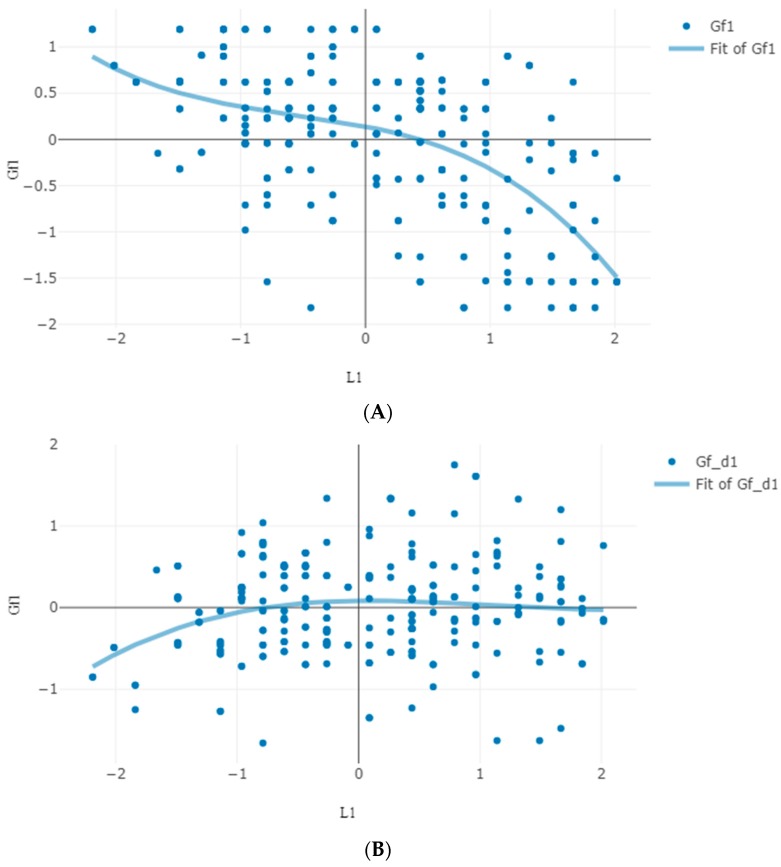
Relations between Gf and L (Panel **A**, R^2^ = 0.39) and change in Gf from first to second testing wave with L (Panel **B**, R^2^ = 0.07).

This model accounted well for the patterns of performance observed (Pearson chi-square (15,526) = 2537.66, *p* = 1.0; likelihood ratio chi-square (15,526) = 773.224, *p* = 1.0; entropy = 0.88). The probability of moving to a higher level was higher (0.54) than staying to the same level (0.46) (odds to progress was 1.17). Of the various covariates, only L exerted a significant influence on transition (2.33, *p* < 0.0001; odds = 10.24) (see Study 1, Model 3, in Supplementary Materials). Overall then, in line with the third prediction about cognizance, higher scores in L at the start were associated with transition to a higher cognitive level. It is noted, however, that despite this overall relation, the cubic relation between cognitive attainment and the product of age by social likeability suggests that early in age (from late childhood to adolescence) it is more likely to change when L is lower rather than higher than latter in age (after the age of 12 years). To model this relation, age was included as a covariate in the model, in addition to the personality scores. Indeed, in this model, individuals with higher L scores were 4.28 times more likely (1.46, *p* < 0.002) to stay at the initial level rather than transition to a higher level as compared to individuals with lower likeability scores. This might imply that at the transition from childhood to adolescence lower likeability scores reflect a stricter and more accurate cognitive self-representation, implying more advanced transition possibilities. Figure 4 illustrates this pattern of relations from first to second testing: Panel A shows the negative relation between Gf and L (R^2^ = 0.39); panel B shows that higher L scores associated with increases in Gf from first to second testing (expressed as the difference between first and second testing scores) especially at initially lower cognitive ability levels (R^2^ = 0.07). 

Normally, the findings above imply that changes in personality are associated with the state of one’s cognitive ability. To explore this possibility, the model above was inverted so that possible cognitive influences on personality changes would be captured. In this model, personality scores on the four dimensions at each testing wave were associated to two categories, staying and changing, and Gf attainment was used as the covariate to specify if personality changes across waves are affected by the initial stage of Gf. Indeed, as expected based on the results above, there was a rather small, but significant, effect of initial cognitive attainment on personality: those starting higher on Gf were more likely to evidence personality changes both from first to second and from first to third testing (1.03, *p* < 0.002; odds = 2.80); lower personality scores were associated with higher likelihood for staying unchanged from first to third testing (−1.58, *p* = 0.02; odds = 0.20) (see Study 1, Model 4, in Supplementary Materials). 

In conclusion, the patterns of relations described above are in line with predictions. L, par excellence, at an earlier time did predict cognitive change and the state of cognition did predict changes in personality. Three interpretations may be given to this combination of patterns. A cognitive explanation would suggest that individuals starting lower in cognition have more room for change. Personality factors, being negatively associated with cognitive attainment, reflect, to some extent, that individuals obtaining extreme scores on personality dimensions are more likely to change cognitively because of their distance from cognitive ceiling. A personality explanation would suggest that individuals high in likeability are involved in a positive loop motivating them to advance cognitively to sustain their positive self- or social image. Finally, a moderate degree of psychoticism is related to higher cognitive achievements. A third interpretation would integrate the two interpretations above into one: changes in self-monitoring and self-regulation processes associated with the transition from rule- to principle based-thought tune cognitive functioning and self-presentations so that they more accurately reflect each other. The studies below will further highlight these relations. 

## 3. Study 2: Transition from Rule- to Principle-Based Thought and the Big Five Factors

This study covered rule-based and principle-based thought, involving participants from 7 through 17 years of age. Study 1 above involved only fluid inferential cognitive processes and personality. This study involved, additionally, executive processes, such as attention control and working memory, and crystallized cognitive processes, such as mathematical reasoning. The translated Croatian version of the 50-item IPIP Big Five inventory [58] was given at the beginning of the second testing. Involving the Big Five Factors allows a more refined mapping of personality processes. Participants were examined twice by the cognitive tests and once by the personality test. Thus, this study may help differentiate possible influences of personality on changes in Gf from the possible effects of executive processes. 

Customizing our general predictions to the present study, we would expect the following patterns of relations: First, the Big Five Factors are hierarchically organized into the stability and plasticity factors which relate to the GFP. Second, according to the first of the predictions stated in the introduction, the g and the GFP are related. Third, executive cognitive processes would relate more to the plasticity factors in personality; fluid and crystallized cognitive processes would relate more to the stability factors. Finally, according to our fourth prediction in introduction, stability factors would impede, but plasticity factors would facilitate development of inferential abilities. 

### 3.1. Method

#### 3.1.1. Participants

A total of 438 right-handed participants from 7 through 17 years of age (206 male) were involved. At first testing, they were 7.92- (15, 12 males), 8.53- (68, 39 males), 9.33- (61, 34 males), 10.73- (21, 5 males), 11.39- (53, 30 males) 12.73- (38, 18 males), 13.30- (52, 28 males), 14.77- (20, 12 males), 15.41- (42, 19 males), 16.61- (35, 17 males), and 17.29-years-old (35, 18 males), respectively. These participants were all native speakers of Croatian and lived in Zagreb, Croatia’s capital. They were students in Croatian public schools and, thus, SES is about equally represented in each age group. 

#### 3.1.2. Tasks

All cognitive tests used here are described in Žebec, Demetriou, and Kortla-Topić [59]. Specifically, the *MID-KOGTESTER1* was used to test speed of processing, selective, and divided attention. This is a computer-based test battery that contains eight cognitive tests. For processing speed, participants responded to the appearance of stimulus (six same color Xs) by lifting their finger from a resting key to touch the target as fast as possible. In choice reaction tasks, participants responded to one of four target stimulus by pressing the appropriate (one of four) response key. For attention control, a Stroop-based task was used: participants responded to the ink color of congruent and incongruent of color words denoting the same or a different color. The Stroop effect, which is the difference in RT between incongruent and congruent tasks, is regarded as a measure of selective attention [60,61]. The *divided attention* (DA) task demanded simultaneous responding to two different tasks on the two panels, where the stimuli were presented in fast succession (50 to 250 ms). Task 1 was a simple reaction time task from the SRT-LH test. Task 2 is an object size classification task from the OSC test, made in the form of two-choice RT task [60]. Participants were asked to respond to Task 1 with the left hand on Panel 1, and on to Task 2 with the right hand on Panel 2. The difference between RT on Task 2 performed together with the Task 1 and RT on Task 2 performed separately was used as a measure of divided attention [61]. Test-retest reliability (across the two testing waves) was high, varying between 0.7 (CRT-C) and 0.85 (DA).

Working memory was addressed by the forward (FDS) and backward (BDS) digit span tasks included in the WISC-III test and extended with two items in FDS and one item in BDS (in order to increase discriminability of older age groups). Test-retest reliability across the two testing waves was satisfactory both for the FDS (0.69) and the BDS (0.66).

Mathematical reasoning was addressed by tasks examining the ability to execute arithmetic operations in combination to each other, algebraic reasoning, and proportional reasoning. Items in each domain scaled along four levels. In the arithmetic tasks, participants were asked to specify the operations missing from simple arithmetic equations: One (e.g., 5 * 3 = 8), two (e.g., {4 # 2} * 2 = 6), three (e.g., {3 * 2 # 4} @ 5 = 7), and four operations (e.g., {5 @ 2} o 4 = {12 $ 1} * 2) were missing from the items of each level. The algebraic reasoning tasks required to specify one or more unknowns in an equation (e.g., *a* + 5 = 8, specify *a*; *u* = *f* + 3; *f* = 1; specify *u*; if (*r* = *s* + *t*) and (*r* + *s* + *t* = 30), specify *r*; when is true that {*L* + *M* + *N*} = {*L* + *P* + *N*}? for the four levels, respectively). In proportional reasoning, the four levels required to grasp relations between the following: (i) fully symmetrical and equivalent ratios (e.g., ½ to 3/6); (ii) equivalent but not obviously symmetrical ratios (e.g., 2/6 to 3/9); (iii) ordered pairs with two corresponding terms multiple of one another (e.g., 2/5 to 3/7); (iv) pairs without corresponding terms (e.g., 5/12 to 3/8). In terms of the cycles of development specified in the introduction, the two lower levels of these batteries are primarily related to the two phases of the rule-based concepts. Levels three and four addressed the two phases of the principles cycle, respectively. This battery was found to have good psychometric and developmental properties in several studies [26]. In the present sample *discriminability* (average index of difficulty of 35 tasks is 0.52 and Ferguson’s Δ is 0.98) and *reliability* were high (Cronbach α = 0.92, split-half = 0.95).

Raven’s standard progressive matrices involve five sets of matrices of increasing complexity. Based on Rasch scaling of performance on each of the 60 matrices, four levels were formed, each involving 15 matrices. From easy to difficult, matrices in the first group, require grasping the pattern underlying figures varying along a single dimension. In the second group, two familiar and obvious dimensions (e.g., shape, size, background, etc.) would have to be integrated. In the third group, matrices require deciphering and integrating critical dimensions through systematic search and transformation of the features involved. For instance, it is the double of …, it goes by one more, etc. Finally, in the fourth group, matrices require deciphering multiple dimensions by grasping the thread underlying several transformations of figures and integrating into complementary general principles. Level 1 addresses abilities of the second phase of the representational cycle. Levels 2 and 3 address abilities associated with the two phases of rule-based thought, respectively. These were the levels represented in the Raven-Like test used in Study 1. Level 4 addresses abilities of first level of principle-based thought.

The translated Croatian version of the 50-item IPIP Big Five inventory included 50 items, 10 for each of the Big Five Factors. This inventory was highly reliable (Cronbach’s alpha = 0.83).

The correlations between reasoning tasks were very high (0.5–0.7). Correlations between personality measures were lower but significant (all but one 0.2–0.3). Correlations between cognitive and personality measures varied according to personality dimension: they were positive and moderate but mostly significant in the case of E (circa 0.2) and negative in the case of C (circa −0.2); the rest varied between 0–0.2 (see correlations in Table S2, in the Supplementary Materials). 

### 3.2. Results

**Figure 5:**
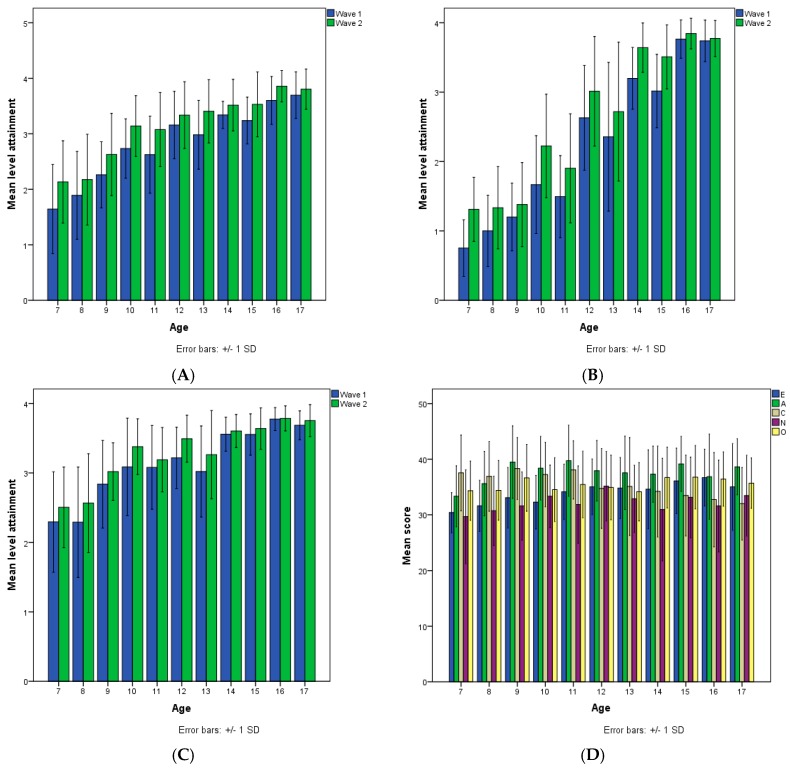
Mean level attainment in arithmetic, algebraic, and Raven reasoning (Panels **A**–**C**) and personality (Panel **D**) as a function of age and testing wave. (**A**) Arithmetic ability; (**B**) Algebraic reasoning; (**C**) Raven; (**D**) Personality traits.

*Development.*Figure 5A–C shows the developmental pattern of cognitive processes as a function of age at first and second testing wave. There were very large changes across all cognitive processes. Overall, in the cognitive domain, children progressed from modal level 1 at 7–8 years of age to modal level 3 at 16–17 years of age, F (10, 421) = 136.09, partial η^2^ = 0.76. Moreover, there was significant progress across all mental processes and across all age groups, F (1, 427) = 269.76, η^2^ = 0.39, from first to second testing. The significant interactions between age and domain, F (30, 1281) = 13.96, η^2^ = 0.25, and age and testing wave F (10, 427) = 4.24, η^2^ = 0.09, as well all three factors, F (30, 1281) = 2.66, η^2^ = 0.06, indicated that the degree of change across age or wave differed across cognitive processes. It is noted that different domains spurted and consolidated at different age phase. It can be seen that arithmetic reasoning spurted from 7 to 11 years of age, indicating, that it is basically a rule-based acquisition; algebraic reasoning demonstrated very little change from 7 to 11 years, but developed very fast between 11 and 14 years, obviously reflecting its principle-based origins; interestingly, performance on Raven matrices developed in two spurts, one from 8 to 10, and another from 13 to 15 years, indicating that it involves a rule-based component and a principle-based component as expected. These patterns are informative for the cognition-personality relations to be presented below.

In concern to personality, the main effect of age, F (10, 425) = 1.92, *p* < 0.05, partial η^2^ = 0.04, personality dimension, F (4, 422) = 37.45, *p* < 0.0001, partial η^2^ = 0.26, and their interaction, F (40, 1700) = 2.78, *p* < 0.0001, partial η^2^ = 0.06, were significant (see Figure 5D). Overall, scores in A exceeded and scores in N lagged behind all other factors, perhaps reflecting the two poles of social likeability, respectively. However, differences varied with age: E increased systematically from 7 to 16 years; A increased from 7 to 9 and stabilized; conscientiousness was basically steady from 7 to 10 and then decreased throughout the remaining period, resembling the inverse relation between age and L observed in Study 1; N increased from 7 to 10 and then wavered; O wavered throughout, with two noticeable spurts between 8 and 9 and 13 and 14. Overall, increases in personality scores occurred from 7 to 10 years and decreases occurred in adolescence, possible reflecting the differential effect of acquiring rule-based and principle-based thought. The models below will highlight this developmental intertwining between cognitive and personality processes. 

#### 3.2.1. Personality Structure and g-GFP Relations

A first set of models examined the organization of the personality factors, involving three scores parceling the ten items addressed to each factor. These models showed that a three-level hierarchical model was superior to any other model tested. In this model, the first-order factors for A, C, and N were related to a second-order stability factor and the first-order factors for E and O were related to a second-order plasticity factor; these second-order factors, in turn, were related to a third-order GPF. To specify if g and the GFP are related, the approach used in Study 1 was employed here. In sake of this aim, the cognitive factors were also included in the model. Specifically, this model involved the following first-order factors for performance on cognitive processes at the second testing wave which was close in time to the measurement of personality: a factor standing for processing speed was related to two RT scores requiring perceptual discrimination where no interference was involved; a factor standing for attention control was related to three scores addressed to divided and selective attention; a factor standing for working memory was related to forward and backward digit span; a factor standing for Gf was related to the five scores attained on the five sets of Raven matrices; a factor standing for mathematical reasoning was related to the mean performance attained on arithmetic, algebraic, and proportional reasoning tasks; these four cognitive factors were related to a second-order cognitive factor. For personality, a mean score for each of the Big Five was involved; as above, A, C, and N were related to a factor standing for stability and E and O were related to a factor standing for plasticity; these two factors were related a second-order GFP. Following the approach adopted in Study 1, in a first model, the correlation between the second-order cognitive factor and the GFP was constrained to be 0, (χ^2^ (162) = 622.23, CFI = 0.90, *p* < 0.001, RMSEA = 0.081, AIC = 298.23); in a second model, these two factors were left free to correlate, (χ^2^ (161) = 600.28, CFI = 0.91, *p* < 0.001, RMSEA = 0.079, AIC = 278.28). The difference between the two models was significant, Δχ^2^ (1) = 21.95, *p* < 0.001, reflecting the fact that the two factors were significantly correlated (0.35). This model is fully presented in Supplementary Materials, Study 2, Model 1. This relation dropped but stayed significant after partialling out the effect of age from each measure-factor relation (0.26). Additionally, this relation stayed in the same range when the general cognitive factor was broken into an executive efficiency factor, related to speed and attention control (0.36) and a representational efficiency factor, including working memory and the two reasoning factors (0.31). These findings are in agreement with our second prediction. 

#### 3.2.2. Personality Mediation

A second set of models tested how cognitive processes from first testing wave influenced personality measures one year later and how personality measures influenced cognitive measures at second testing. Specifically, this model involved the four first-order cognitive factors involved in the model above; the speed and attention control factors were associated to an executive efficiency factor; the working memory, mathematical reasoning, and Gf factors were associated to a factor standing for representational and inferential power (RIP). It is noted that this factor is narrower than psychometric g because it does not include speed and attention control measures, but broader than psychometric Gf because it includes, in addition to psychometric Gf (the Raven test), working memory and mathematical reasoning; this model also included the three of the Big Five factors, i.e., A, C, and N, related to stability of personality, the α-factor, and the other two of Big Five factors, i.e., E and O, associated with plasticity of personality, the β-factor. The following structural relations were built in this model. On the one hand, each of the Big Five factors was regressed on the two first wave second-order factors standing for processing and representational efficiency, to capture how first wave cognitive processes influenced personality dimensions. On the other hand, each of the two second wave cognitive factors were regressed on the two personality factors, the α-factor and the β-factor. Corresponding measurement errors across waves were correlated. To examine equivalence of factors across waves, the model was tested under the constraint that corresponding measurement-factor relations were free to vary or equal across testing waves. 

**Figure 6:**
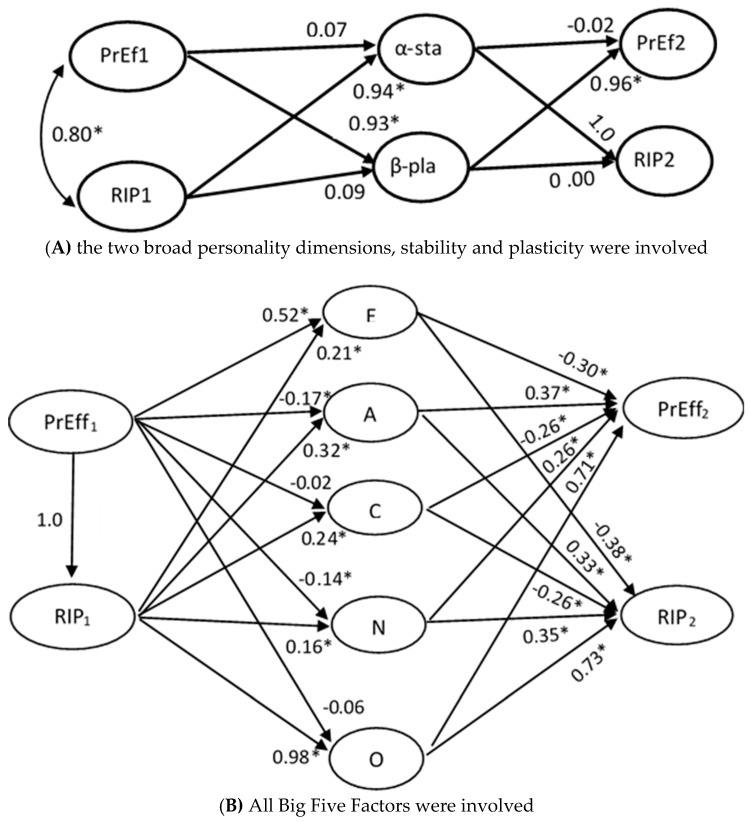
Cross-lagged models (panel A and B) of the mediation of personality factors of stability (α-factor) and plasticity (β-factor) between processing efficiency (PrEf) and representational and inferential power (RIP) at first and second testing wave (1 and 2). Note: Models A and B are fully presented in Supplementary Materials, Study 2, Models 2 and 3, respectively).

Notably, the fit of the constrained model, χ^2^ (544) = 1839.87, *p* > 0.001, RMSEA = 0.074, model AIC = 751.87, was acceptable and better than the fit of the unconstrained model, χ^2^ (536) = 2060.84, *p* > 0.001, RMSEA = 0.081, model AIC = 988.84. This is the model shown in Figure 6A. The pattern of relations was very interesting: the α-factor, stability, was very strongly related to RIP (0.94) and very weakly to processing efficiency (0.07); plasticity, the β-factor, demonstrated the inverse pattern of relations: it was weakly related to RIP (0.09) but strongly to processing efficiency (0.93). This pattern of relations was replicated in the effects of personality on cognitive performance at second wave: plasticity influenced processing efficiency (0.96) but not stability (−0.02); stability influenced RIP (1.0). A possible explanation of these very high relations is in order here. Probably, our cognitive tasks, being very carefully scaled developmentally, are sensitive to cognitive variation that is relatively masked in psychometric tests where standardization in reference to individual differences suppresses developmental variation. These relations were further probed in a model where personality factors were individually involved (Figure 6B). In this model, processing efficiency at first testing was significantly related to E (0.52), A (−0.17), and N (−0.14); RIP at first testing was significantly related to all Big Five factors; notably, however, only its relation with O was very high (0.98); the rest were moderate (~0.2–0.3). Interestingly, all Big Five factors related significantly with both cognitive factors at second testing. Again the relations of all but O with each of these two cognitive factors varied circa 0.3; the relation of O with both cognitive factors was very high (~0.7); attention is drawn to the negative relation of C with both second-testing cognitive factors. It is suggested, in agreement with our third prediction, that personality interacts developmentally with cognition at both the level of individual Big Five factors and the level of more general factors representing stability and plasticity, with A and O to be the factors leading these two general personality orientations. 

Latent transition analysis was again used to specify how personality is related to cognitive transition to a higher level. In this analysis level attainment on the three mathematical reasoning batteries and RPM at the two testing waves was used in the model. Specifically, level attainment at first testing on each of these cognitive dimensions was related to one category and level on each dimension at second testing was related to a second category. There were two classes in each category, those staying at the initial class and those changing, either moving to a higher class (category 1) or regressing to a lower class (category 2). At a first test of the model, attention control, working memory, and stability (α-factor), and flexibility (β-factor) of personality were used as covariates. This model fit the data very well (Pearson chi-square (199,864) = 4015.07, *p* = 1.0; likelihood ratio chi-square (199,864) = 1236.704, *p* = 1.0; Entropy = 0.88; AIC = 6439.75). The probability of moving to a higher level was good (0.46) although lower than staying to the same level (0.54) (odds to progress was 0.84). All four covariates significantly affected transition: the effect of attention control was quite large (4.85, *p* < 0.001; odds = 128.14); the effect of working memory (1.06; *p* < 0.001; odds = 2.9) and plasticity of personality (β-factor; *p* < 0.04; odds = 2.11) (0.74) was significant and considerable; the effect of stability (α-factor) (−0.76; *p* < 0.005; odds = 0.47) was significant, but negative. In concern to personality, these effects suggest that individuals high in flexibility were 2.11 times more likely to transition to a higher level from first to second testing than to stay at their first testing initial level. On the contrary, individuals high in stability were 0.47 times more likely to stay at their initial level than progress to a higher level. To further probe the origins of these effects, a second model was tested where the two personality general factors were dropped as covariates and conscientiousness and openness were used in their place. This model fit the data equally well, although slightly lower than the first model (entropy 0.89; AIC = 6455.66). Notably, the effect of conscientiousness on transition was significant and negative (−0.70, *p* < 0.0001; odds = 0.57) and the effect of openness was significant and positive (0.96, *p* < 0.04; odds = 2.61). In agreement with our fourth prediction, it is suggested that personality is involved in cognitive change, with some dimensions impeding and some dimensions facilitating cognitive development (see Model 4, Study 2, Supplementary Materials). 

To examine if the involvement of personality in cognitive change varies with developmental phase, the LTA model above was applied separately on two age groups, 7–11-year and 12–16-year olds. Some interesting differences between these age groups were observed. First, the probability to transition to a higher state in the younger age group was limited (odds = 0.14) compared to the older age group (odds = 1.13). This is understandable given that all reasoning domains but arithmetic were principle-based acquisitions which consolidate in adolescence rather than in childhood. Moreover, there were noticeable differences in the factors associated with transition in each age phase. In the younger age group, only working memory (1.23, *p* < 0.001; odds = 3.34) and plasticity affected transition significantly (1.45, *p* < 0.07; odds = 4.26). The pattern in the older age group was very similar to the pattern found in the total sample: attention control (2.82, *p* < 0.01, odds = 16.76), working memory (1.00, *p* < 0.0001, odds = 2.72), and plasticity (0.57, *p* < 0.10, odds = 1.78) significantly affected transition and stability exerted a negative effect (−0.72, *p* < 0.05, odds = 0.49) on transition. 

Therefore, it is suggested that personality is differentially involved in cognitive development. In different developmental cycles different processes relate to change. In rule-based thought, working memory and plasticity catalyze the transition to principle-based thought. When already in principle-based thought, stability decelerates change, competing with factors which accelerate change. 

## 4. Study 3: Cognition, Cognizance, Personality, Emotional Intelligence, and Academic Performance: What Is the Go-between?

This study examined a wide range of cognitive, personality, emotional intelligence, and school performance processes from 10 to 20 years of age. Specifically, cognitive abilities included reasoning in several domains (inductive, quantitative, causal, spatial, and social reasoning along a range of developmental levels) and cognizance (self-evaluation of own’s own performance and self-representation in the domains above); personality included the Big Five Factors; emotional intelligence was examined both as a trait (self-representations about emotional characteristics) and as an ability (understanding and regulating emotions); finally, information on participants’ school performance in Greek and mathematics was obtained. Therefore, this study may show how cognitive, personality, and emotional processes interact from late childhood to early adulthood and how they contribute to academic achievement. 

Customizing initial predictions to the present study allows testing the following: first, cognition-personality relations would hold, even when likeability effects are removed as possible sources of these effects; second, according to the second and third prediction stated in introduction, given the self-representational nature of personality and emotional intelligence, cognizance would occupy a central role, operating as the bridge between cognition and personality. Finally, according to the fifth prediction, academic performance, being the outcome of cognitive, personality, and motivational processes, would depend on all of them. 

### 4.1. Method

#### 4.1.1. Participants

A total of 247 participants were examined. They came from fifth primary school grade (45, 25 females; mean age = 10.7 years), first (47, 27 females; mean age = 12.5 years), third (42, 25 females; mean age = 14.9 years), and fifth secondary school grades (33, 22 females; mean age = 16.7), and university (80, 55 females; mean age 20.3 years). These participants had urban residence, living in Nicosia, the capital of Cyprus, and they were representative of the total urban population, dominated by middle-class families. 

#### 4.1.2. Tasks

*Cognition.* Cognition was examined by a part of a cognitive development test addressed to several domains [53]. For the present purposes, the following domains were involved: *inductive* (fluid) reasoning was addressed by six Raven-like matrices of varying complexity as specified in Study 1 (levels, ii, iii, and iv); *scientific reasoning* was examined by a combinatorial thinking task (specify all possible combinations in the order of drawing several differently colored balls from a bag) and hypothesis testing by properly mapping hypotheses of varying complexity with relevant patterns experiments; *quantitative reasoning* was examined by algebraic (e.g., specify x if x = y + z and x + y + z = 30; when is true that L + M + N = L + P + N) and proportional reasoning tasks (e.g., specify how may girls and boys there are in a classroom where we have 30 children and five out of six are girls); *spatial reasoning* was examined by a paper folding task addressed to mental rotation and two coordination of perspectives tasks (specify the level of liquid in a tilted bottle and the direction of a an object hanging in a track moving at various inclinations); *social reasoning* was examined by two tasks requiring understanding of social intentions and consequences of social actions (Cronbach’s alpha = 0.88). 

Cognizance was examined by two tests. First, participants self-evaluated their performance on a task from each of the domains included in the cognitive battery above (Raven-like matrices, hypothesis testing, algebraic reasoning, paper folding, and social reasoning). Each of these self-evaluation scores was standardized in relation to the performance score obtained on the respective task to reflect accuracy of self-evaluation (Cronbach’s alpha = 0.54). 

Second, they answered a self-rating inventory involving three types of self-descriptions: (i) domain-specific abilities related to the domains above (e.g., I can easily decipher how to solve a mathematical problem; I can easily discriminate between evidence related and not related to an event; I easily orient myself in a new city; I can grasp the hidden intentions of others); (ii) *cognizance abilities* related to cognition, emotions, and social behavior (e.g., I know where I am strong and where I am weak; I am able to know my body states (thoughts, emotions); I can easily shift between activities; I can appear calm when I am angry; I can focus on a task even if tired); (iii) *general processing efficiency* (e.g., I am very fast in learning new concepts; I am fast in finding the solution of a problem; I can remember verbatim big chunks of text; I easily keep phone numbers in memory). Scores for self-representation in mathematical, causal-scientific, spatial, and social reasoning, visual and phonological memory, self-control, and ability to know oneself were used in the analyses below (Cronbach’s alpha = 0.90).

Personality was examined by the Greek version of the Big Five Personality Inventory. This test addressed two or three facets for each of the Big Five: achievement and order for C; anxiety and self-consciousness for N; extroversion and introversion for E; altruism, sensitivity to others, and agreeableness for A; and intellect and openness for O (Cronbach’s alpha = 0.87).

Emotional intelligence was examined by two tests. First, trait emotional intelligence was examined by a self-rating inventory (scale 1–5) addressed to *knowledge about emotions* (e.g., I know why my emotions change, I control my emotions, when I am in a good mood I have many new ideas), *sensitivity in recognizing and emitting verbal and non-verbal emotional signals* (e.g., I am aware of the emotional signals I send to others, I recognize someone’s emotions on his phase), *style of reacting to emotionally loaded events* (When I get high school marks I am not affected, I am not moved by a new nice present; I am indifferent to praise), and *emotional self-regulation* (e.g., I control my emotions) (Cronbach’s alpha = 0.81). This inventory was based on the emotional intelligence scale developed by Schutte [62]. 

Emotional intelligence ability was examined by two tests. The first involved three sections, each addressed to a different aspect of understanding emotions: First, participants rated the degree (1–5) of involvement of several emotions (anger, sadness, joy, disgust, fear, and surprise) in several real-life episodes (obtaining high school marks, failing and winning in sports competitions, impasse in solving a problem, preparing for exams, setting goals for the school year, dealing with a dilemma). The scores given to the most relevant emotion was used in each case (e.g., success-joy, failure-sorrow). Second, this test also examined the ability to specify the emotions involved in a combination of mental states, such as pleasure and expectation, joy and acceptance, sorrow and disappointment, joy and calmness, etc. Nine pairs were given which were scored on a pass-fail basis. The sum of this test was used in the analysis. Third, participants were asked to write three stories about two persons shifting from one emotional state to another (e.g., from being calm and careless to being fearful and anxious; one first feels satisfied, then pleased, then enthused and, finally, surprised and proud). Each story was scored on a three-point scale (0, 1, and 2 for irrelevant, partly and fully relevant. The mean score of performance on the three stories was used in the analysis. This test is based on the test originally developed by Mayer and colleagues [63,64,65]. 

To examine explicit representation of emotions, participants were asked to specify (on 1–5 scale) how much each of 15 descriptions or definitions apply to three emotions (joy, grief, and surprise). The factor score of the first principal component of a factor analysis applied on the ratings for each emotion was used in the analyses below (Cronbach’s alpha = 0.69). 

School performance was evaluated by school teachers in two subjects: Greek and mathematics. Teachers were asked to rate (1–7 scale) each of their students in several domains: learning complex concepts, learning speed, originality, understanding and using complex concepts, interest, and actual performance in the subject (Cronbach’s alpha = 0.98). School performance measures were not available for university students (N = 80). 

The correlations between measures varied in the fashion of the previous studies. Measures addressed to the same construct correlated moderately to high (circa 0.3 to 0.6); correlations across constructs varied between 0 to circa 0.3 (see correlations in Table S3, in the Supplementary Materials). 

### 4.2. Results

#### 4.2.1. Developmental Patterns

**Figure 7:**
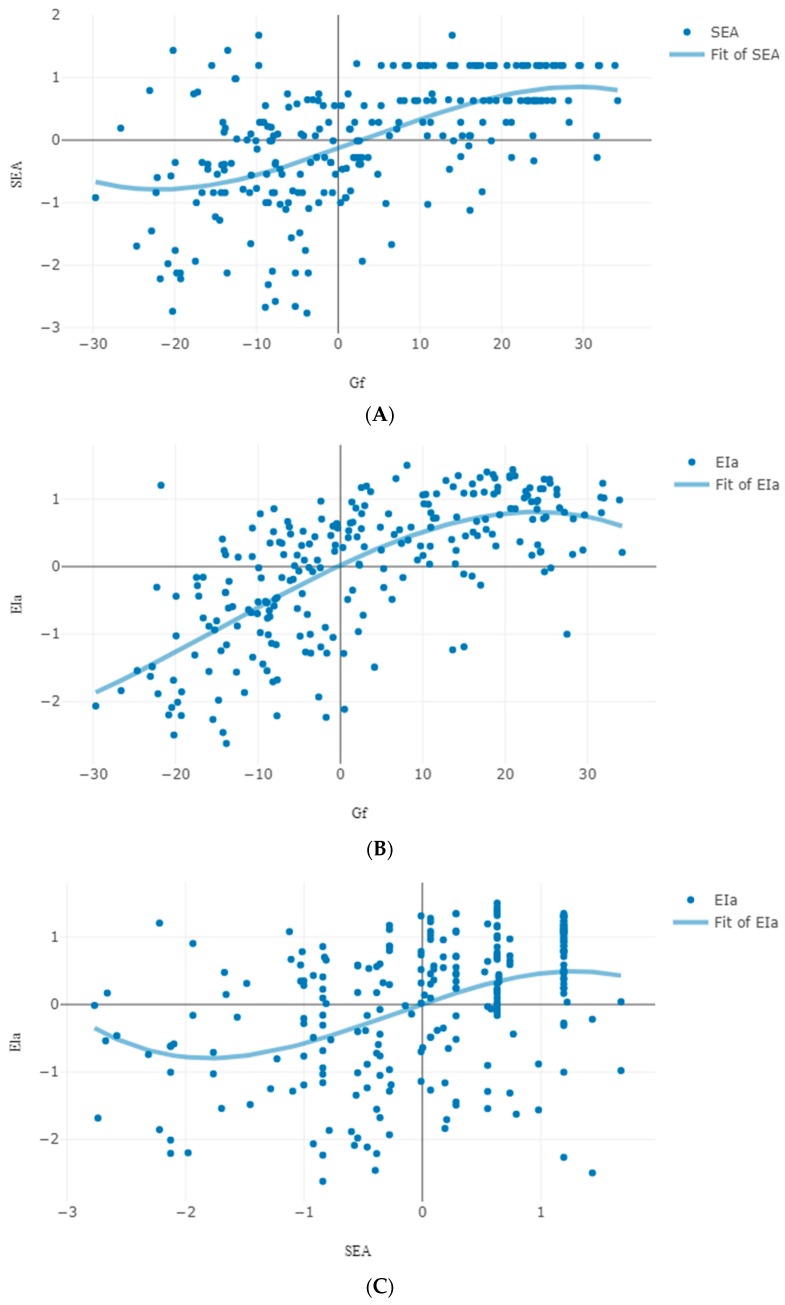
Relations between Gf and SEA (self-evaluation accuracy) (Panel **A**, R^2^ = 0.38), Gf and EIa (ability to understand and define emotions) (Panel **B**, R^2^ = 0.49); and SEA and EIa (Panel **C**, R^2^ = 0.05).

Developmental patterns vary as a function of process. Expectedly, all cognitive abilities developed systematically throughout the age span covered. Individual univariate ANOVAs showed that the effect of age on (i) cognitive performance, F (4, 242) = 83.70, *p* < 0.0001; η^2^ = 0.58; (ii) self-evaluation accuracy, F (4, 242) = 31.36, *p* < 0.0001; η^2^ = 0.34; and (iii) understanding emotions was always strong, F (4, 242) = 36.86, *p* < 0.0001; η^2^ = 0.38, reflecting an almost linear increase in each ability with age. Obviously, these three aspects of cognitive ability are strongly intertwined in development. Figure 7 illustrates the covariation of the product of age with the factor score of the first principal component abstracted from each of the three sets of scores. 

Relations between age and the various traits differed from above. Specifically, self-representations in cognizance (which is a second-order self-representation) decreased systematically with age. As a result, the main effect of age was non-significant, F (4, 242) = 0.65, *p* > 0.05; η^2^ = 0.01, but the interaction of age with self-representation type was significant, F (4, 242) = 7.14, *p* < 0.0001; η^2^ = 0.11. 

Similarly, the main effect of age on personality was non-significant, F (4, 242) = 2.09, *p* > 0.05; η^2^ = 0.03). However, the main effect of personality dimension, F (4, 242) = 71.03, *p* < 0.0001; η^2^ = 0.54), and the age x personality interaction were significant, F (4, 242) = 3.61, *p* < 0.0001; η^2^ = 0.06). These effects reflected two main trends: N was lower than the rest but increased with age; E was high but decreased; A decreased from 10 to 14 and increased thereafter, topping the rest after 16 years. C decreased from 10 to 14 and rose thereafter; O decreased systematically from 10 through 16 and rose from there to 20 years. In emotional intelligence, the main effect of age was again non-significant, F (4, 242) = 1.21, *p* > 0.05; η^2^ = 0.02; however, processes did differ significantly, F (2, 242) = 323.22, *p* < 0.0001; η^2^ = 0.73) and they (marginally) differentially related with age, F (2, 242) = 1.73, *p* < 0.08; η^2^ = 0.03. The cognitive aspects of trait EI (understanding of emotions and regulation of emotional signals wavered with age, but self-representation of emotional stability tended to decrease (Figure 7). In conclusion, expansion of cognitive ability with age was differentially reflected in various aspects of self-representation and personality with some varying with states of ability and some becoming stricter or more conservative. The models to be presented below will shed light on these relations.

#### 4.2.2. Relations between Processes

The multiplicity of measures used in this study provides a rich basis for studying the relations between the various processes measured. Thus, two different approaches were used. First, exploratory graph analysis was used to map the organization of processes. Second, structural equation modeling was used to specify their relations.

*Exploratory Graph Analysis (EGA)*. Exploratory graph analysis is part of a new area called network psychometrics (see [66]), which focuses on the estimation of undirected network models (i.e., Markov random fields [67]) to psychological datasets. EGA can show if measures form nodes that connect with each other into clusters standing for underlying latent variables. Golino and Demetriou [68] showed that EGA is more accurate than other methods, including confirmatory factor analysis, to reveal the dimensions underlying performance on various cognitive test batteries under a variety of test and sample conditions. They suggested that EGA may be the method of choice to uncover underlying dimensions of behavior or ability in fields of study where clear theory specifying constructs and their relations is not yet available. Structural equation methods may then be used to validate EGA findings and more exactly specify the direction of relations between constructs. 

**Figure 8:**
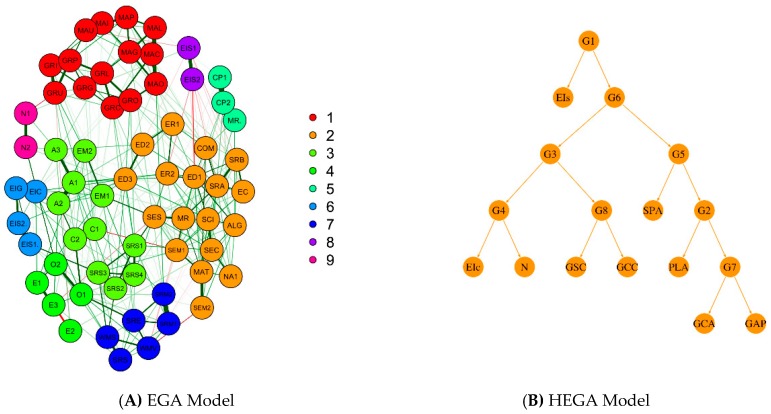
Network of abilities and processes as abstracted by exploratory graph analysis (Panel **A**) and hierarchical organization of clusters as abstracted by hierarchical exploratory graph analysis (Panel **B**). Note: clusters represent general academic performance measures (1: GAP), general cognitive ability (2: GCA), general self-representation of social competence cluster (3: GSC), plasticity of personality (4: PLA), spatial reasoning (5: SPA), knowing one’s own emotions (6: EIc), self-representation of mental efficiency (7: GCC), emotional stability (8: EIs), and neuroticism (9: N) from the Big Five, respectively.

It may be noted here that each of the Big Five Factors emerged as a separate cluster when only the scores obtained from the Big Five Inventory were used in the analysis. Figure 8A shows the best-fitting EGA model, χ^2^ (1791) = 4074.84, CFI = 0.98, RMSEA = 0.072, applied on the full set of scores obtained in this study. Clusters are shown in different colors. It may be seen that there are three very broad clusters and several narrower ones. The first broad cluster included all academic performance measures (GAP). The second was a cognition cluster including most of the cognitive and all self-evaluation accuracy (SEA) and the ability to understand emotions scores (EIa). Therefore, this is a very powerful cluster standing for general cognitive ability (GCA). Notably, there was also a very broad self-representation of the social competence cluster (GSC), which included self-representations of causal and social reasoning, A and C from the Big Five, and handling exchange of emotional information from EIt. Plasticity of personality, including all E and O measures, emerged as a separate cluster (PLA). Clusters 5, 6, 8, and 9 are specific, standing for spatial reasoning (5: SPA), knowing one’s own emotions (6: EIc), self-representation of mental efficiency (7: GCC), emotional stability (8: EIs), and neuroticism (9: N) from the Big Five, respectively. 

The hierarchical organization of these factors was explored by hierarchical exploratory graph analysis (HEGA). HEGA uses hierarchical random graphs to specify the hierarchical organization of the various latent factors. Hierarchical random graphs were developed by Clauset, Moore, and Newman [69] as a probabilistic technique to detect hierarchies in network structures. It works by combining a maximum-likelihood approach with a Monte Carlo sampling algorithm to generate artificial networks with a given hierarchical structure. By using this approach, the space with all possible dendrograms whose probability is proportional to the likelihood that they generated the observed data is analyzed. A consensus dendrogram is obtained by using a Markov chain Monte Carlo (MCMC) algorithm in an estimated hierarchical random graph, containing only the dendrogram features that appear in the majority of the sampled models. Hierarchical random graphs can be combined with exploratory graph analysis to investigate how different latent factors are organized in a hierarchy. 

In this paper we adopted a three-step approach. First, data was iteratively analyzed by EGA until all factors related to at least two items and the resulting structure was verified by CFA to fit the data well. Second, based on this structure, a factor score for each factor was computed for all participants; these factor scores were again fed in EGA to estimate a network of the relationships between the variables. Finally, the network obtained in the previous step was used as input to the hierarchical random graphs technique, available in the *igrpah* package, until a consensus hierarchical network was obtained. This network is derived by weighting the hierarchical features by their likelihood based on a process akin to Bayesian model averaging. This is dendrogram model that may most likely generate the observed data [70]. 

In line with structural equation modeling, the HEGA approach can be used to identify structural relations in multivariate data. However, while in the latter the relationships need to be specified by the researcher, in the former this is done automatically. Additionally, the HEGA approach is used to see how variables are connected in a hierarchical organization only. In SEM, the structure can have any form, not just hierarchical. Thus, both techniques can be seen as complimentary to each other, HEGA being an exploratory approach and SEM a confirmatory one. 

This organization is shown in Figure 8B: it may be seen that the nine factors above are organized in two major systems, one capturing the procedural cognitive aspects of the mind (**M***_c_*) and the other the self-representational, social and emotional aspects of the mind (**M***_s_*). The **M***_c_* system is grounded on general cognitive ability and academic performance, which integrate into a common block standing for thinking and learning (G7); this intertwines with the α-factor, plasticity of personality, to form a higher level, flexibility in cognition and learning (G2); finally, flexible cognition and learning integrate spatial cognition into a higher level (G5). **M***_s_* is grounded on two branches: the first (G8) involves self-representation of general social (GSC) and cognitive competence (GCC); the second (G4) involves N from the Big Five and EIc; these two branches unite into a higher level (G3) which, together with **M***_c_* (G5) form a level where the two systems merge into a higher level common (G6). This, together with emotional stability merge at the top in what Wechsler would call the personality as whole g**P**. 

*Testing the effect of likeability on structural relations*. The findings above suggested that cognition, personality, emotional intelligence, and academic performance are related. To decompose their relations at several levels, three classes of models were tested. The first class of models examined if these relations are shaped by likeability rather than their sharing of common processes [29]. In sake of this aim a likeability scale was formed. This scale included 14 items explicitly probing individuals to specify how they score on several positive characteristics in cognition (e.g., I have a strong memory, I am fast in understanding), personality (e.g., I am organized, I am bright, I am honest), and emotional intelligence (e.g., I know what others feel by just looking at them). The reliability of this scale was high (Cronbach’s alpha = 0.74). Notably, the correlations between this scale and age (−0.35) and Gf (−0.32) were similar in magnitude and direction to the corresponding correlations of Eysenck’s L scale observed in Study 1, suggesting that likeability decreases with age or cognitive ability.

To examine the possible effect of this scale on the relations between factors, a model was built which included three cognitive factors (Gf, understanding, and specifying emotions), three cognizance factors (self-evaluation accuracy, self-representation in specific domains, and self-representation of self-knowing ability), the Big Five Factors, and three traits of emotional intelligence (i.e., self-awareness about emotions, emotional stability, and recognition and management of emotional signals). The three cognitive factors were related to one general cognition factor (g); the three cognizance factors were related to another (COGN); C, A, and N were related to one factor (α-stability) and O and E to another (β-plasticity); the three EI trait factors were related to another factor; these last three factors were related to a common higher-order personality factor (GFP). At a first test of the model, the correlations between these three higher-order factors (g, COGN and GFP) were constrained to be 0, (χ^2^ (1020) = 2267.32, CFI = 0.991, *p* > 0.001, RMSEA = 0.071, model AIC = 227.32. At a next test, these correlations were left free to be estimated, (χ^2^ (1017) = 2161.07, CFI = 0.992, *p* > 0.001, RMSEA = 0.068, model AIC = 127.08. The difference in the fit of the two models was highly significant (Δχ^2^ (3) = 106.25, *p* < 0.001), reflecting the fact that all three correlations were significant (R_g,COGN_ = 0.53; R_g,GFP_ = 0.24; R_COGN,GFP_ = 0.64). Finally, this last model was tested after partialling out the effect of the likeability scale from the relations of each cognizance, personality, and emotional intelligence measure with the factor it was related to, (χ^2^ (1030) = 2098.11, CFI = 0.992, *p* > 0.001, RMSEA = 0.065, model AIC = 227.32. Although still good, the fit of this model was not better than the second model above. This reflected the fact that the correlations between the three higher order factors were still significant (R_g,COGN_ = 0.67; R_g,GFP_ = 0.42; R_COGN,GFP_ = 0.62); noticeably, the correlation between g and the GFP increased rather than dropped in spite of partialling likeability out. Obviously, the relations between these three factors are genuine rather than an artefact of likeability (Study 3, Model 1, Supplementary Materials). 

**Figure 9:**
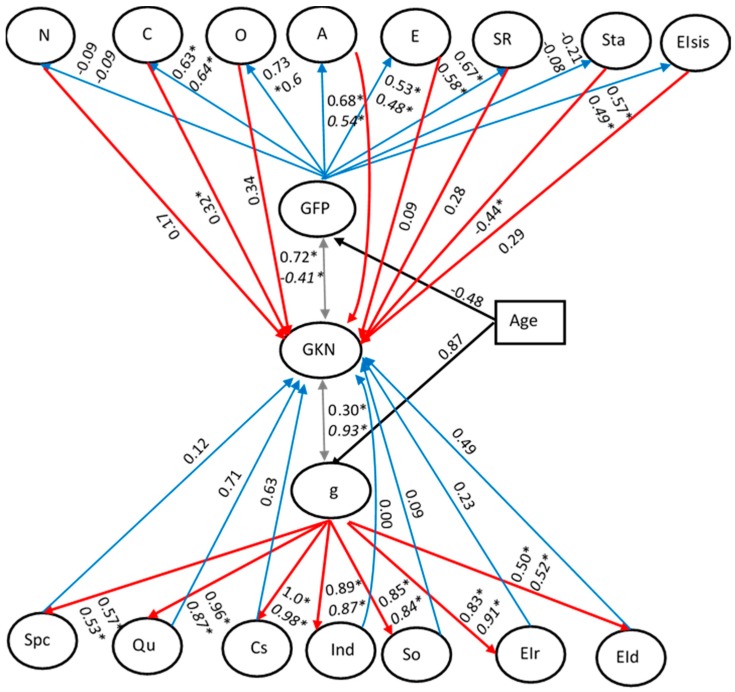
The “cognition ⟶ personality” (blue arrows) and “personality ⟶ cognition” (red arrows) mediation models showing how cognizance mediates between cognition and personality. Note. The first and second number pair come from the “cognition ⟶ personality” and the “personality ⟶ cognition” model, respectively. The grey arrows connecting g, cognizance (COGN), and the GFP should be read as either pointing upwards (the “cognition ⟶ personality” model, first number of each pair) or downwards (the “personality ⟶ cognition” model, second number of each pair). Symbols Spc, Qu, Cs, and Ind stand for spatial, quantitative, causal, and inductive reasoning; symbols EIr and EId stand for the ability to understand, and specify emotions, respectively; the symbols N, C, O, A, and E stand for the Big Five Factors; the symbols SR, Sta, and EIsis stand for self-awareness about emotions, emotional stability, and recognition and management of emotional signals, respectively. Asterisks indicate significant relations (see complete models in Supplementary Materials, Study 3, Models 2 and 3).

*Specifying cognizance mediation.* The second class of models tested the assumed mediation role of cognizance. Specifically, models in this class tested how, if at all, cognizance mediates between cognitive and personality processes, carrying experiences from mental processing to personality and emotional dispositions and vice versa. These models included the following first-order cognitive factors: spatial, quantitative, causal, inductive, and social reasoning defined by performance on the tasks described above (see method); two factors capturing emotional intelligence as an ability (i.e., association of different emotions with corresponding real-life situation and specification of characteristics of different emotions); all seven factors were regressed on a second-order Gf factor. Also, there were three factors for cognizance (i.e., SEA, related to self-evaluation accuracy; SR_d_, related to self-representation about these domains; SR_sk_ related to self-representation of self-knowing ability, which were regressed on a second-order cognizance factor (COGN). Finally, there were eight factors for personality and emotional intelligence traits (i.e., one factor for each of the Big Five factors, associated with the facet scores included in each factor—see method—and self-awareness about emotions, emotional stability, and recognition and management of emotional signals); these eight factors were regressed on a common second-order factor standing for GFP. To examine the mediation role of cognizance, in the “cognition ⟶ personality” model, the COGN factor was regressed on Gf and the residuals of each of the seven domain-specific cognitive factors; the GFP was regressed on COGN. Thus, this model captures how cognizance mediates the effects of general cognitive ability and the specific processes represented by each domain-specific factor to the personality and emotional intelligence factor. The fit of this bottom-up model was very good, χ^2^ (1251) = 2327.93, CFI = 0.997, *p* > 0.001, RMSEA = 0.060, model AIC = −174.07 (Study 3, Model 2, Supplementary Materials). In the “personality ⟶ cognition” model, the COGN factor was regressed on GFP and the residuals of each of the personality and the emotional intelligence trait factors; the Gf factor was regressed on the COGN factor; thus, this model captures how cognizance mediates the possible effects of GFP and each of the specific personality and emotional intelligence traits on cognitive ability. Although also good, the fit of this top-down model was slightly weaker than the fit of the “cognition ⟶ personality” model, χ^2^ (1252) = 2506.55, CFI = 0.996, *p* > 0.001, RMSEA = 0.064, model AIC = 2.47. Two further models were tested: one assumed that Gf is the mediator between COGN and personality, χ^2^ (1254) = 2367.07, CFI = 0.997, *p* > 0.001, RMSEA = 0.061, model AIC = −140.93; the other assumed that GFP is the mediator between cognition and cognizance, χ^2^ (1254) = 2564.70, CFI = 0.997, *p* > 0.001, RMSEA = 0.066, model AIC = 56.70 (Study 3, Model 3, Supplementary Materials). Both models were weaker than the “cognition ⟶ personality” model, although the first of them fit better than the ”personality ⟶ cognition” model. Figure 9 shows the “cognition ⟶ personality” model; the values of the “personality ⟶ cognition” model are also shown for comparison purposes. 

Altogether, these models suggest that there is a strong flow of influences running across cognitive and personality systems. The supremacy of the cognitive over the personality mediation models suggests that cognitive mechanisms operate as stronger relay centers than personality mechanisms. If a direction of the flow of influences would have to be chosen, the supremacy of the “cognition ⟶ personality” cognizance mediation model indicates that experiences of mental processing project onto cognizance which carries them forward to personality and emotional functioning. It may be seen in Figure 9 that, in addition to Gf (0.30), quantitative reasoning (0.71), and the ability to specify different emotions (0.49) exerted strong influences on cognizance. In turn, cognizance exerted strong effects on GFP (0.72) and also four of the Big Five (C, O, A, E) and two of EI traits (SR, EIsis) (all > 0.5). In the “personality ⟶ cognition” model, all but one of the eight Big Five and EI traits exerted significant effects on cognizance with C, A, and emotional stability to be in the lead. Notably, the effect of the GFP on COGN was negative (−0.41), reflecting the already substantiated involvement of likeability in the relations between personality and self-representation and self-evaluation. Attention is drawn to the cross-sectional nature of the present study. Ideally, longitudinal results would be needed to disentangle when in development cognizance carries effects from cognitive processes to personality and when it carries effects from personality dispositions to mental processes. 

**Figure 10:**
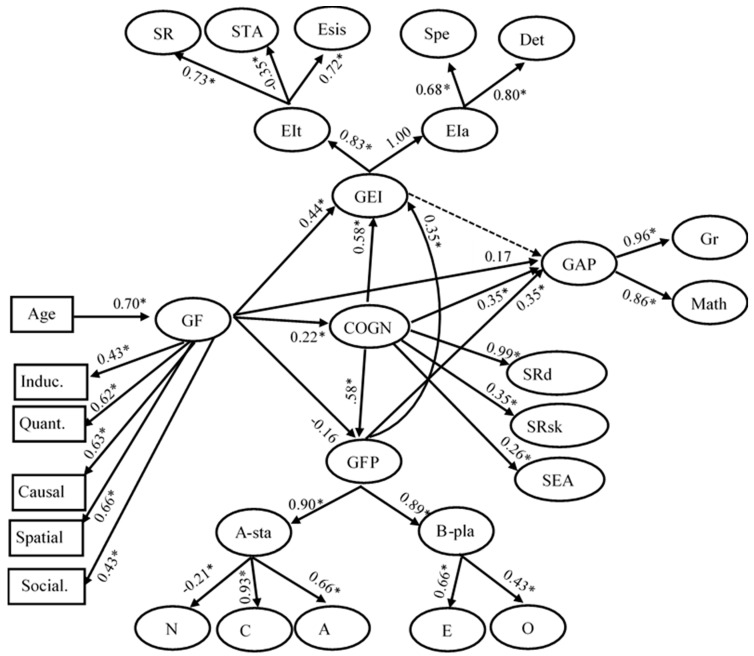
The second model of cognitive abilities, personality, emotional intelligence and school performance. χ^2^ (1103) = 1701.49, *p* < 0.05, CFI = 0.994, RMSEA = 0.058 (0.052–0.06), AIC = −404.51. Note. Symbols are specified in Figure 9. Additionally, the symbols Gr and Math stand for school performance in Greek and mathematics, respectively; GAP stands for general academic performance; the symbols SRd, SRsk, and SEA stand for self-representation in specific domains, self-knowledge and self-regulation, and self-evaluation, respectively; the symbols EIa and EIt stand for ability and trait emotional intelligence, respectively; A-STA and B-PLA stand for the stability plasticity factors of personality, respectively (see complete model in Supplementary Materials, Study 3, Model 4).

*Accounting for academic performance.* The third class of models aimed to specify how academic performance relates to the various processes studied. The best fitting model is shown in Figure 10. This model involved the following first-order factors: Gf, related to mean performance on each of the cognitive domains; the three cognizance factors above (SEA, SR_d_, and SR_sk_) were related to a second-order factor standing for cognizance (COGN). Three of the Big Five factors, N, C, and A were related to the α-factor, stability, and E and O were related to the β-factor, plasticity of personality; in turn, these two second-order factors were regressed on a third-order factor standing for the GFP. The three factors capturing emotional intelligence traits and two factors capturing emotional intelligence as an ability were regressed on their corresponding second-order factors (EIt and EIa); these factors were regressed on the general emotional intelligence factor (GEI). Finally, academic performance was related to the means of performance in Greek and mathematics (GAP). The following structural relations were built into the model: Gf was regressed on age, COGN was regressed on Gf, GFP was regressed on Gf and the residual of COGN; GEI was regressed on Gf and the residuals of COGN and GFP; GAP was regressed on Gf and the residuals of COGN, GFP, and GFI factor. This model implements the assumption suggested by the bottom-up mediation model presented above that cognizance mediates between cognitive ability, on the one hand, and personality and emotional intelligence, on the other hand. Using only the Gf factor as such in all relations and the residuals of the other factors assumes that each factor, at a specific level in the hierarchy, involves a fundamental component of mental processing and, additionally, other processes specific to the levels intervening between general cognitive ability and the specific factor concerned. Residualizing these intervening factors purifies them, technically speaking, from components specific to the other factors already used. The fit of this model was very good, χ^2^ (1103) = 1701.49, *p* > 0.001, CFI = 0.994, RMSEA = 0.058. 

This model is complementary to the network and the hierarchical models presented above in that it highlights the (statistically) causal relations between the various clusters. Noticeably, cognizance was a central hub in the system: it *was* affected by Gf (0.22) but it *did* affect the GFP (0.58), GEI (0.58) and academic performance (0.35); Gf also significantly affected GEI (0.44), academic performance (0.17) and, non-significantly, GFP (−0.17); the negative direction of this relation reflected the influence of A (see below); academic performance was also affected, equally, by both GFP (0.35) and GEI (0.35). To examined possible differences in the effects of the α- and the β-factor of personality on GAP, the GFP was abolished and GAP was regressed on both personality factors. Notably, the effect of plasticity, the β-factor (0.81) was much higher than the effect of stability, the α-factor (0.42). 

One might note that the relation between Gf and GAP was lower than is often reported in the literature. Indeed, in the present data this relation increased to 0.40 in a model where only the Gf and the school performance factors were involved; further, it rose to 0.56 when Gf and the cognizance factors were associated to a single GCA factor. Regressing cognizance on Gf (0.60) and GAP on Gf (0.18) and the residual of cognizance (0.63) showed that the later relates more strongly with GAP than the former. Along the same line, a model involving only the personality and the academic performance factors indicated that personality effects on school performance, in addition to the GFP as such (0.26), originated from C (0.33), A (−0.19), and O (0.15). 

This model was tested in a two-group analysis including participants from 10 to 14 years in one group and participants from 16 to 20 years in another group. All measurement-factor relations were constrained to be equal across the two groups; the factor-factor relations were left free to vary across the groups to examine if relations between factors change with age. The fit of this model was good, χ^2^ (2003) = 3231.85, CFI = 0.997, *p* > 0.001, RMSEA = 0.071, model AIC = −774.35. Of the various relations, only three noticeable differences between these two age groups emerged: on the one hand, the effect of Gf on the GEI in the younger group (0.33) was significantly higher (z = 3.22, *p* < 0.01) than in the older group (−0.10); on the other hand, the effect of Gf on cognizance (0.19 vs. 0.29 for the younger and older participants, respectively; z = 1.06, *p* > 0.05) and the effect of cognizance on the GFP (0.55 vs. 0.93; z = 2.41, *p* < 0.01) were larger in the older group. This pattern of age differences indicates that early in adolescence, a period of transition from rule- to principle-based thought, cognitive changes influence EI; later, when cognitive ability gets stabilized and cognizance becomes sharper and more accurate, self-representations and self-characterizations in personality also become more accurate, and sometimes stricter than early in development. As discussed below, these relations are generally in line with predictions.

In conclusion, the results of this study suggested, in line with predictions, that the relations between general factors of cognition, personality, and emotional are present even when likeability is statistically removed. Cognizance was a central core underlying these relations. Finally, school performance was affected by both, cognitive and cognizance factors, but also personality factors, conscientiousness in particular. 

## 5. Conclusions

The implications of our findings may be better evaluated if some unique characteristics of the three studies are highlighted. Altogether, the three studies included (1) a wide age range, from 7 to 20 years, which is crucial for cognitive and personality development; (2) a wide range of cognitive (information processing, executive control, reasoning, and self-awareness) and personality processes (Eysenck’s factors, the Big Five Factors, and emotional intelligence), uncovering relations that would not be observed otherwise; (3) three different countries (Greece, Croatia, and Cyprus), providing cross-cultural validity to the findings; and (4) longitudinal measures highlighting developmental relations within individuals rather than different age cohorts. 

*Structure.* Altogether, the three studies suggest, in line with the first prediction, that cognition and personality are distinct, but related, at several levels. At a basic level, the network and hierarchical models in Study 3 suggested that some processes in each system interlock it with the other system: mental plasticity (O in particular) is personality’s envoy in cognition; self-concept is cognition’s envoy in personality. At a higher level, all three studies showed that the relation between g and the GFP was significantly and substantively different from 0 in the models. This finding suggested that mental power is projected into personality, regardless of age or social likeability. We trust that the great scholars mentioned in the introduction, Allport, Wechsler, and Jensen, would be pleased to see that cognition (*g*) and personality (GFP) merge into gP. It is reminded that the general factor in each discipline, g in intelligence and GFP in personality, is disputed within each field as a technical artefact of measurement. The present studies suggest a Godelian restoration of both in the context of a higher-order factor, gP, which captures the substance of both: general processing, representational, and inferential efficiency (cognition) that is expressed into a person’s dispositional efficiency in handling his or her interactions with the world (personality). Cognizance is the central mechanism shared by the two general factors. This mechanism translates experiences from cognitive and social interactions with the world into values of self-worth, confidence, and self-efficacy, rendering them complementary manifestations of these two aspects of efficiency. These values set the range of variation across personality dimensions, such as each of the Big Five Factors or broader dimensions, such as stability and plasticity. All three studies showed that the inverse influences may also hold: personality strength as captured by several personality dimensions, such as the feeling that one can cope with the unexpected (O) or one possesses resources and strategies to deal with complex life demands (C), may project into mental functioning, motivating the individual to engage in mental processing; or, if negative, this feeling may drive the individual to disengage from mental processing, resulting into missed opportunities for learning and development. 

These findings are relevant to the dispute about the existence and nature of GFP. Some authors dispute its very existence, claiming that it reflects social desirability running through self-ratings [71] or measures that carry same-factor components across unrelated factors [72] rather than any other actual psychological mechanism. Others suggest that its presence varies depending upon the level of measures used to specify relations. For instance, it emerges in self-ratings but not in multi-rater nested data [73] and its strength increases with increasing hierarchical level of the dimensions involved [74]. The studies presented here suggest a comprehensive interpretation of these seemingly divergent patterns of evidence. For one thing, the GFP survived even when the effect of desirability was statistically partialled out (Study 3). For another, when present, desirability is an indicator of cognizance processes shared by cognition and personality rather than as noise to be removed. These processes rescale self-ratings towards the stricter end of a personal self-evaluation scale along with intellectual growth; hence, the ubiquitous negative relation between indices of intellectual growth and various indices of desirability (Study 1). Naturally, in line with the findings above [74], GFP strengthens with increasing hierarchical level of the dimensions involved because cognizance processes are by definition second- or higher-order processes: i.e., they apply to task-specific cognitive or personality processes, although they may emerge from processes where task-specific processes and self-regulation processes merge in the same task, as in attention control processes (Study 2). 

The so-called emotional intelligence was not an autonomous dimension. Its cognitive components were absorbed by the inferential system and its social components aligned with personality factors standing for dispositions underlying social interaction. These findings confirm recent research showing that emotional intelligence is probably synonymous with the processes captured by the GFP [75]. However, Study 3 suggested that cognitive processes activated in dealing with emotions do have a role in cognition-personality relations that go beyond cognition: they contribute special experiences in the formation of cognizance which are projected in personality. 

*Development.* Relations between cognition and personality vary with development, depending on the representational possibilities and the behavioral and social needs of successive developmental phases. According to our second prediction, self-representations improve with age and liaise between cognition and personality with increasing accuracy and refinement. Study 3 showed that the accuracy of self-evaluations of actual cognitive performance improved systematically from 9 to 20 years, along with improvements in cognitive ability. In fact, alignment of all aspects of self-representation with each other and cognitive ability tightened with age, suggesting that cognitive processing, be it object- or socially-emotionally oriented, becomes inseparable from the various aspects of cognizance. 

According to the third prediction, cognizance would function as the central hub between all other functions involved. This was indeed the case, as suggested by Study 3. In fact, this role expressed itself in several seemingly unrelated trends captured by the other studies. Specifically, at a first glance, it would be paradoxical that Eysenck’s likeability would be such a powerful developmental factor, as found by Study 1: its decrease with age was an accurate index of cognitive development. Obviously, L stands up, among the other personality factors, as a strong self-representational agent: with development it scales down to reflect increasing accuracy in self-representation and self-evaluation. Obviously, a decrease here does not imply a loss of ability; it implies adjustments in self-representations of both cognitive and social characteristics, along with cognitive improvement. These seemingly contrasting trends give a developmental dimension to Socrates’ epistemic insight: “The only thing I know is that I know nothing” is not just an attainment of philosophical minds. It is a developmental construction inherent in cognitive change which is gradually integrated into the development of personality. Additionally, these trends indicate that high L scores, when persisting in adulthood, may be a frozen remnant of developmental processes that are highly active in late childhood. A developmental interpretation of this remnant might invoke the developmental equivalent of average IQ of 100 characterizing the majority of adults. According to this interpretation, mean IQ corresponds to late rule-based thought [5]. Thus, high levels of L in the general population express the general population’s modal cognitive developmental level. 

According to the fourth prediction, some aspects of personality have a privileged relation with various aspects of cognition. This was a standard finding across all three studies. On the one hand, plasticity, the β-factor, proved to be an integral component of the mind. In Study 2, the β-factor as such, O in particular, contributed to the likelihood of longitudinal change, especially in adolescence. Notably, the inverse relation was also observed. That is, cognitive change did raise the likelihood for changes in personality. Study 2 showed that plasticity did carry some deep-rooted trends associated with attentional control, which, at the surface may not seem related to a top-tier level of personality functioning, such as the β-factor. The relations uncovered by the model shown in Figure 6 (Study 2) suggested that an advantage in processing efficiency at a given time translated into increased plasticity one year later. In Study 3, the β-factor aligned with the cognitive, rather than with the personality, system. On the other hand, some aspects of the α-factor, C in particular, negatively related to the likelihood of cognitive developmental change (Study 2). In contrast, in line with the expected paradox, C did relate positively with academic performance. One might assume that this apparent paradox simply reflects the double role of C in actual functioning in real life. The first: individuals high in C are slower in cognitive change, perhaps settling lower at their final cognitive level because they are conservative in facing challenges inducing change; this is the advantage of individuals high in O and E. It is interesting that this effect was observed at the age period related to the acquisition of principle-based thought rather than earlier. Thus, one might assume that C interferes with cognitive change related to the acquisition of a more open, suppositional style of thought rather than the more systematic rule-based thought of childhood. The second: this very seeming weakness provides an advantage in realms of activity where self-discipline, order, and systematicity pay off in the long run. Obviously, school learning and academic performance is one such realm, par excellence. More research is needed to map and more precisely specify these effects. 

The patterns above suggest a more balanced picture of cognition-personality relations than the relations suggested by the investment model summarized in the introduction. The investment model assumes that cognitive ability is a capital that may or may not be profitably invested by personality investing agencies [49,50,51]. This economic interpretation of the human mind assumes a segregation between cognition and personality that does not do justice to its integrity which drives the developing individual to establish an optimally balanced relation with the environment. Our findings suggest that cognitive development is an adaptive constructive process that includes a mental (representational and inferential), an interactive (dealing with the physical, the cultural, and social environment), and a motivational component (the will to pursue specific actions given their relative demand on mental resources and their relative value for ongoing interaction needs). All three components are inter-dependent and changes in any one of them would cause changes in the others, expanding the adaptive possibilities of the individual. Under this model, developmental changes in mental processes would cause changes in their expression in interactive and motivational tendencies as much as the results of interactive and motivational engagement with the environment may feedback to mental activities causing their further development. 

The loops of causal interactions between these three components may vary in development, depending upon the cognizance possibilities of successive developmental cycles. For instance, the relatively superficial self-monitoring possibilities of the realistic representations cycle would not generate the necessary representational material that would allow the preschool child to deploy a representational change strategy in response to negative social or action feedback or a behavioural or motivational change strategy in response to the evaluation of interactive possibilities associated with alternative representations of a situation. In childhood, the consolidation of rule-based thought and the turn of cognizance from perceptual to mental and personality processes enable children to more systematically conduct themselves in a world of obligations and expectations, where different contexts demand different behaviors. At this period of life, however, the nascent self-monitoring and self-representation possibilities still lack an overall evaluation system that would allow to place values on different experiences and actions. Hence, the inflation of self-value judgments, reflecting this developmentally nascent sense of mental and behavioral power. Later in adolescence, emergence of principle-based thought and increasing resolution of self-knowing causes a more conservative approach to self-representation which integrates epistemic recognition of the limits of one’s own mental power with the limitations of one’s positive characteristics. This allows relatively fluid multi-directional interactions between the three components in adolescence, for those individuals who do enter principle-based thought. 

*Limitations*. The findings of the three studies suggest several limitations in our understanding of cognition-personality relations in development. First, self-ratings expressing self-representations, which is typical in personality research, may be relatively valid with late rule-based thought or later. Self-ratings require (i) relatively explicit self-representations that may be subjectively dimensionalized and (ii) mapping these subjective dimensions onto a scale varying along a certain metric specified by the researcher. Both abilities are not present before late rule-based thought. In fact, it is only with principle-based thought, in adolescence, that persons possess an elaborate self-evaluation ability and a refined self-concept that they may use to specify their cognitive, emotional, personality, and behavioral characteristics [76]. Even then, the desirability may always linger, even if technically removed as done in Study 3, thus distorting cognition-personality relations [77]. Therefore, change in the state of personality dimensions and in the relations between cognition and personality from childhood to adulthood observed by research [6,10,43,44] may reflect changes in the accuracy of self-monitoring and self-evaluation as much as actual changes in the relations between mental and personality processes. Further research is needed that would compare cognition-personality relations as emerging from actual social interactions with relations emerging from self-ratings as it was done here. 

A second limitation is concerned with the precise cognition-personality relations at successive developmental cycles. For instance, what are the ideal values of each of the Big Five at each cognitive developmental phase or cycle? Are the Big Five Factors relevant for the cycles of episodic or realistic representations? It might be the case that at these early periods of life an ideal mastery of executive processes with control of emotion are more relevant to successful development than dispositions such as agreeableness or openness [5,7]. What level of openness or conscientiousness goes with rule-based or principle-based thought? It might be the case that rule-based thought goes well with C whereas principle-based thought goes well with O. Study 2 suggested that this is possible. It is noted here that we may not fully understand how cognition-personality relations vary later in life unless we understand how they relate early in life when changes occur in both. For instance, recent research suggested that O continues to associate with cognitive ability in old age and that, with conscientiousness, slow cognitive decline [78,79]. The present studies provided only limited information relevant to these questions because the methods used for mapping personality dispositions were designed for adults; therefore, they are minimally sensitive to developmental variations. We hope, however, that raising these questions will direct future research to generate better answers than we obtained here. 

## Supplementary Materials

The following are available online at http://www.mdpi.com/2079-3200/6/4/51/s1. Supplementary 1—Tables presenting correlations and statistics of the variables used in the models presented in the three studies: Table S1. Correlations, means, SD of the variables used in Study 1 (total sample); Table S2. Correlations, means, SD of the variables used in Study 2 (total sample); Table S3. Correlations, means, SD of the variables used in Study 3 (total sample). Supplementary 2—Codes used in building the various models presented in the three studies.

## References

[1] Wechsler D. (1935). The Range of Human Abilities.

[2] Allport G.W. (1937). Personality: A Psychological Interpretation.

[3] Cattell R.B. (1965). The Scientific Analysis of Personality.

[4] Jensen A.R. (1998). The G Factor: The Science of Mental Ability.

[5] Demetriou A., Spanoudis G., Rosen M. (2017). Mind and Intelliegence: Integrating developmental, psychometric, and cognitive theories of human mind. Challenges in Educational Measurement—Contents and Methods.

[6] Caspi A. (2005). Personality development: Stability and change. Annu. Rev. Psychol..

[7] Rothbart M.K. (2011). Becoming Who We Are: Temperament and Personality in Development.

[8] Ackerman P.L. (2018). The search for personality–intelligence relations: Methodological and conceptual issues. J. Intell..

[9] Chamorro-Premuzic T., Furnham A. (2006). Personality and Intellectual Competence.

[10] Ziegler M., Danay E., Heene M., Asendorpf J., Buhner M. (2012). Openness, fluid intelligence, and crystallized intelligence: Toward an integrative model. J. Res. Personal..

[11] Carroll J.B. (1993). Human Cognitive Abilities: A Survey of Factor-Analytic Studies.

[12] Flynn J.R. (2009). What Is Intelligence: Beyond the Flynn Effect.

[13] Hunt E. (2011). Human Intelligence.

[14] Demetriou A., Spanoudis G., Kazi S., Mouyi A., Žebec M.S., Kazali E., Golino H.F., Bakracevic K., Shayer M. (2017). Developmental differentiation and binding of mental processes with re-morphing g through the life-span. J. Intell..

[15] Demetriou A., Makris N., Kazi S., Spanoudis G., Shayer M. (2018). The developmental trinity of mind: Cognizance, executive control, and reasoning. WIREs Cogn. Sci..

[16] Demetriou A., Makris N., Kazi S., Spanoudis G., Shayer M., Kazali E. (2018). Mapping the dimensions of general intelligence: An integrated differential-developmental theory. Hum. Dev..

[17] Eysenck H.J., Eysenck S.B.G. (1969). The Structure and Measurement of Personality.

[18] Eysenck H.J. (1992). A reply to Costa and McCrae. P or A and C—The role of theory. Personal. Individ. Differ..

[19] Eysenck H.J. (1992). Four ways five factors are not basic. Personal. Individ. Differ..

[20] Costa P.T., McCrae R.R. (1992). Four ways five factors are basic. Personal. Individ. Differ..

[21] Rushton J.P., Irwing P. (2009). A General Factor of Personality in 16 sets of the Big Five, the Guilford–Zimmerman Temperament Survey, the California Psychological Inventory, and the Temperament and Character Inventory. Personal. Individ. Differ..

[22] Van der Linden D., te Nijenhuis I., Bakker A.B. (2010). The General Factor of Personality: A meta-analysis of Big Five intercorrelations and a criterion-related validity study. J. Res. Personal..

[23] Harter S. (2015). The Construction of the Self: Developmental and Sociocultural Foundations.

[24] Bandura A. (1986). The explanatory and predictive scope of self-efficacy theory. J. Soc. Clin. Psychol..

[25] Stankov L. (2018). Low correlations between intelligence and Big Five Personality Traits: Need to broaden the domain of personality. J. Intell..

[26] Meyer G.J. (2000). The incremental validity of the Rorschach Prognostic Rating Scale over the MMPI Ego Strength Scale and IQ. J. Personal. Assess..

[27] Van der Maas H.L.J., Dolan C.V., Grasman R.P.P.P., Wicherts J.M., Huizenga H.M., Raijmakers M.E.J. (2006). A dynamical model of general intelligence: The positive manifold of intelligence by mutualism. Psychol. Rev..

[28] Van der Maas H.L.J., Kan K.-J., Marsman M., Stevenson E.C. (2017). Network models for cognitive development and intelligence. J. Intell..

[29] Ferguson E., Chamorro-Premuzic T., Pickering A., Weiss A., Chamorro-Premuzic T., Von Stumm S., Furnham A. (2011). Five into one doesn’t go: A critique of the General Factor of Personality. The Wiley-Blackwell Handbook of Individual Differences.

[30] Hill W.D., Arslan R.C., Xia C., Luciano M., Amador C., Navarro P., Hayward C., Nagy R., Porteous D.J., McIntosh A.M. (2018). Genomic analysis of family data reveals additional genetic effects on intelligence and personality. Mol. Psychiatry.

[31] Demetriou A., Spanoudis G. (2018). Growing Minds: A Developmental Theory of Intelligence, Brain, and Education.

[32] Haier R.J. (2017). The Neuroscience of Intelligence.

[33] Bouchard T.J. (2004). Genetic influence on human psychological traits. Curr. Dir. Psychol. Sci..

[34] Bouchard T.J., McGue M. (2003). Genetic and environmental influences on human psychological differences. J. Nuroboil..

[35] Loehlin J.C., Bartels M., Boomsma D.I., Bratko D., Martin N.G., Nichols R.C., Wright M.J. (2015). Is there a genetic correlation between General Factors of Intelligence and Personality?. Twin Res. Hum. Genet..

[36] Blair C. (2006). How similar are fluid cognition and general intelligence? A developmental neuroscience perspective on fluid cognition as an aspect of human cognitive ability. Behav. Brain Sci..

[37] MacDonald K.B. (2008). Effortful control, explicit processing, and the regulation of human evolved predispositions. Psychol. Rev..

[38] Erdle S., Irwing P., Rushton J.P., Park J. (2010). The general factor of personality and its relation to self-esteem in 628,640 internet respondents. Personal. Individ. Differ..

[39] Demetriou A., Kyriakides L., Avraamidou C. (2003). The Missing link in the relations between intelligence and personality. J. Res. Personal..

[40] Case R. (1992). The Mind’s Staircase: Exploring the Conceptual Underpinnings of Children’s Thought and Knowledge.

[41] Pascual-Leone J. (1987). Organismic processes for neo-Piagetian theories: A dialectical causal account of cognitive development. Int. J. Psychol..

[42] Piaget J., Mussen P.H. (1970). Piaget’s theory. Carmichael’s Handbook of Child Development.

[43] Asendorpf J.B., van Aken M.A.G. (2003). Validity of Big Five Personality Judgments in childhood: A 9 year longitudinal study. Eur. J. Personal..

[44] Lamb M.E., Chuang S.S., Wessels H., Broberg A.G., Hwang C.P. (2002). Emergence and construct validation of the Big Five Factors in early childhood: A longitudinal analysis of their ontogeny in Sweden. Child Dev..

[45] McCrae R.R., Costa P.T., Ostendorf F., Angleitner A., Hřebıčková M., Avia M.D., Sanz J., Sánchez-Bernardos M.L., Kusdil M.E., Woodfield R. (2000). Nature over nurture: Temperament, personality, and lifespan development. J. Personal. Soc. Psychol..

[46] Roberts B.W., Walton K.E., Viechtbauer W. (2006). Patterns of mean-level change in personality traits across the life course: A meta-analysis of longitudinal studies. Psychol. Bull..

[47] Antinori A., Carter O.L., Smillie L.D. (2017). Seeing it both ways: Openness to experience and binocular rivalry suppression. J. Res. Personal..

[48] McIntyre M., Graziano W.G. (2016). Seeing people, seeing things: Individual differences in selective attention. Personal. Soc. Psychol. Bull..

[49] Ackerman P.L., Heggestad E.D. (1997). Intelligence, personality, and interests: Evidence for overlapping traits. Psychol. Bull..

[50] Von Stumm S., Ackerman P.L. (2013). Investment and intellect: A Review and meta-analysis. Psychol. Bull..

[51] Von Stumm S., Chamorro-Premuzic T., Ackerman P.L., Chamorro-Premuzic T., von Stumm S., Furnham A. (2011). Revisiting intelligence–personality associations: Vindicating intellectual investment. Handbook of Individual Differences.

[52] Demetriou A., Christou C., Spanoudis G., Platsidou M. (2002). The Development of Mental Processing: Efficiency, Working Memory, and Thinking. Monogr. Soc. Res. Child Dev..

[53] Demetriou A., Kyriakides L. (2006). A Rasch-measurement model analysis of cognitive developmental sequences: Validating a comprehensive theory of cognitive development. Br. J. Educ. Psychol..

[54] Barrett P.T., Petrides K.V., Eysenck S.B.G., Eysenck H.J. (1998). The Eysenck Personality Questionnaire: An examination of the factorial similarity of P, E, N, and L across 34 countries. Personal. Individ. Differ..

[55] Zuckerman M., Kuhlman D.M., Joireman J., Teta P., Kraft M. (1993). A comparison of three structural models for personality: The Big Three, the Big Five, and the Alternative Five. J. Personal. Soc. Psychol..

[56] Shayer M., Demetriou A., Pervez M. (1988). The structure and scaling of concrete operational thought: Three studies in four countries. Genet. Soc. Gen. Psychol. Monogr..

[57] Muthen B., Asparouhov T. (2011). LTA in Mplus: Transition Probabilities Influenced by Covariates.

[58] Mlačić B., Goldberg L.R. (2007). An Analysis of a Cross-Cultural Personality Inventory: The IPIP Big-Five Factor Markers in Croatia. J. Personal. Assess..

[59] Žebec M., Demetriou A., Kotrla-Topić M. (2015). Changing expressions of general intelligence in development: A 2-wave longitudinal study from 7 to 18 Years of age. Intelligence.

[60] MacLeod C.M. (1991). Half a century of research on the Stroop effect: An integrative review. Psychol. Bull..

[61] Pashler H.E. (1998). The Psychology of Attention.

[62] Schutte N.S., Malouff J.M., Hall L.E., Haggerty D.J., Cooper J.T., Golden C.J., Dornheim L. (1998). Development and validation of a measure of emotional intelligence. Personal. Individ. Differ..

[63] Mayer J.D., Salovey P., Caruso D.R. (2002). MSCEIT User’s Manual.

[64] Mayer J.D., Salovey P., Caruso D.R., Sitarenios G. (2003). Measuring emotional intelligence with the MSCEIT V2.0. Emotion.

[65] Mayer J.D., Salovey P., Caruso D.R. (2004). Emotional intelligence: Theory, Findings and implications. Psychol. Inq..

[66] Epskamp S., Maris G., Waldorp L.J., Borsboom D. (2016). Network psychometrics. arXiv.

[67] Lauritzen S.L. (1996). Graphical Models.

[68] Golino H.F., Demetriou A. (2017). Estimating the dimensionality of intelligence like data using Exploratory Graph Analysis. Intelligence.

[69] Clauset A., Moore C., Newman M.E. (2008). Hierarchical structure and the prediction of missing links in networks. Nature.

[70] Csardi G., Nepusz T. (2006). The igraph software package for complex network research. Inter J. Complex Syst..

[71] Bäckström M., Björklund F., Larsson M.R. (2009). Five-factor inventories have a major General Factor related to social desirability which can be reduced by framing items neutrally. J. Res. Personal..

[72] Ashton M.C., Lee K., Goldberg L.R., de Vries R.E. (2009). Higher order factors of personality: Do they exist?. Personal. Soc. Psychol. Rev..

[73] Danay E., Ziegler M. (2011). Is there really a single factor of personality? A multirater approach to the apex of personality. J. Res. Personal..

[74] Kretzschmar A., Spengler M., Schubert A.-L., Steinmayr R., Ziegler M. (2018). The relation of personality and intelligence—What can the Brunswik symmetry principle tell us?. J. Intell..

[75] Van der Linden D., Pekaar K.A., Schrmer J.A., Vernon P.A., Dunkel C.S., Petrides K.V. (2017). Overlap between the General Factor of Personality and Emotional Intelligence: A meta-analysis. Psychol. Bull..

[76] Demetriou A., Boekaerts M., Pintrich P.R., Zeidner M. (2000). Organization and development of self-understanding and self- regulation: Toward a general theory. Handbook of Self-Regulation.

[77] Bensch D., Paulhus D.L., Stankov L., Ziegler M. (2017). Teasing Apart Overclaiming, Overconfidence, and Socially Desirable Responding. Assessment.

[78] Curtis R.G., Soubelet A. (2015). The Relationship between Big-5 Personality Traits and Cognitive Ability in Older Adults—A Review. Aging Neuropsychol. Cogn..

[79] Ziegler M., Cengia A., Mussel P., Gerstorf D. (2015). Openness as a buffer against cognitive decline: The Openness-Fluid-Crystallized-Intelligence (OFCI) model applied to late adulthood. Psychol. Aging.

