# Supplementary material for: Mind-Personality Relations from Childhood to Early Adulthood
Source: J Intell. 2018 Dec 6;6(4):51. doi: 10.3390/jintelligence6040051 (PMC6480795; doi:10.3390/jintelligence6040051)
Supplement: Supplementary file 1 [file jintelligence-06-00051-s001.zip › Supplementary_1_Tables.docx]

***Supplementary Materials***

Mind-Personality Relations from Childhood to Early Adulthood

**Table S1.** Correlations, means, SD of the variables used in Study 1 (total sample).

| Variables | 1 | 2 | 3 | 4 | 5 | 6 | 7 | 8 | 9 | 10 | 11 | 12 | 13 | 14 | 15 | 16 | 17 | 18 |
| --- | --- | --- | --- | --- | --- | --- | --- | --- | --- | --- | --- | --- | --- | --- | --- | --- | --- | --- |
| 1. age | 1.00 |  |  |  |  |  |  |  |  |  |  |  |  |  |  |  |  |  |
| 2. mat1 | 0.63 | 1.00 |  |  |  |  |  |  |  |  |  |  |  |  |  |  |  |  |
| 3. mat2 | 0.49 | 0.49 | 1.00 |  |  |  |  |  |  |  |  |  |  |  |  |  |  |  |
| 4. mat3 | 0.36 | 0.48 | 0.49 | 1.00 |  |  |  |  |  |  |  |  |  |  |  |  |  |  |
| 5. ded1 | 0.57 | 0.48 | 0.35 | 0.29 | 1.00 |  |  |  |  |  |  |  |  |  |  |  |  |  |
| 6. ded2 | 0.57 | 0.44 | 0.45 | 0.47 | 0.47 | 1.00 |  |  |  |  |  |  |  |  |  |  |  |  |
| 7. ded3 | 0.52 | 0.43 | 0.37 | 0.34 | 0.45 | 0.58 | 1.00 |  |  |  |  |  |  |  |  |  |  |  |
| 8. sci1 | 0.64 | 0.55 | 0.33 | 0.32 | 0.41 | 0.43 | 0.42 | 1.00 |  |  |  |  |  |  |  |  |  |  |
| 9. sci2 | 0.58 | 0.56 | 0.39 | 0.37 | 0.45 | 0.46 | 0.42 | 0.67 | 1.00 |  |  |  |  |  |  |  |  |  |
| 10. sci3 | 0.58 | 0.50 | 0.51 | 0.41 | 0.45 | 0.50 | 0.40 | 0.56 | 0.64 | 1.00 |  |  |  |  |  |  |  |  |
| 11. P1 | 0.11 | 0.03 | 0.00 | 0.02 | 0.05 | 0.13 | 0.06 | 0.16 | 0.13 | 0.12 | 1.00 |  |  |  |  |  |  |  |
| 12. N1 | 0.26 | 0.16 | 0.19 | 0.11 | 0.18 | 0.20 | 0.11 | 0.15 | 0.18 | 0.18 | 0.22 | 1.00 |  |  |  |  |  |  |
| 13. E1 | 0.01 | 0.00 | 0.05 | 0.04 | 0.04 | 0.01 | 0.12 | −0.05 | −0.12 | 0.01 | 0.13 | −0.13 | 1.00 |  |  |  |  |  |
| 14. L1 | −0.51 | −0.41 | −0.31 | −0.22 | −0.39 | −0.36 | −0.30 | −0.45 | −0.37 | −0.38 | −0.49 | −0.53 | 0.00 | 1.00 |  |  |  |  |
| 15. P2 | 0.15 | 0.11 | 0.08 | 0.03 | 0.14 | 0.15 | 0.13 | 0.26 | 0.21 | 0.23 | 0.72 | 0.18 | 0.11 | −0.49 | 1.00 |  |  |  |
| 16. N2 | 0.18 | 0.07 | 0.23 | 0.09 | 0.13 | 0.19 | 0.14 | 0.14 | 0.11 | 0.11 | 0.10 | 0.76 | −0.16 | −0.34 | 0.11 | 1.00 |  |  |
| 17. E2 | 0.01 | 0.09 | 0.08 | 0.14 | −0.04 | 0.04 | 0.10 | 0.01 | −0.05 | 0.03 | 0.22 | −0.10 | 0.79 | −0.07 | 0.16 | −0.15 | 1.00 |  |
| 18. P2 | −0.50 | −0.42 | −0.39 | −0.24 | −0.40 | −0.39 | −0.37 | −0.43 | −0.40 | −0.40 | −0.40 | −0.49 | −0.06 | 0.85 | −0.49 | −0.39 | −0.09 | 1.00 |
| Mean | 12.6 | 3.04 | 3.47 | 3.64 | 2.21 | 2.61 | 2.98 | 1.82 | 2.65 | 2.81 | 2.25 | 9.40 | 17.38 | 12.50 | 2.52 | 8.46 | 17.99 | 11.27 |
| S.D. | 1.70 | 1.21 | 0.84 | 0.71 | 0.87 | 0.80 | 0.63 | 1.17 | 1.25 | 1.17 | 2.11 | 4.48 | 3.34 | 5.71 | 2.24 | 5.02 | 3.19 | 6.06 |

Note. The symbols mat, ded, and sci stand for Raven-like matrices, deductive, and scientific reasoning; the symbols P, N, E and L stand for Eysenck’s factors of psychoticism, neuroticism, extroversion, and likeability-lying, respectively. Numbers indicate testing wave.

**Table S2.** Correlations, means, SD of the variables used in Study 2 (total sample).

| Variables | 1 | 2 | 3 | 4 | 5 | 6 | 7 | 8 | 9 | 10 | 11 | 12 | 13 | 14 | 15 | 16 | 17 | 18 | 19 | 20 |
| --- | --- | --- | --- | --- | --- | --- | --- | --- | --- | --- | --- | --- | --- | --- | --- | --- | --- | --- | --- | --- |
| 1. FDS_1 | 1.00 |  |  |  |  |  |  |  |  |  |  |  |  |  |  |  |  |  |  |  |
| 2. BDS_1 | 0.69 | 1.00 |  |  |  |  |  |  |  |  |  |  |  |  |  |  |  |  |  |  |
| 3. FDS_2 | 0.70 | 0.62 | 1.00 |  |  |  |  |  |  |  |  |  |  |  |  |  |  |  |  |  |
| 4. BDS_2 | 0.59 | 0.64 | 0.69 | 1.00 |  |  |  |  |  |  |  |  |  |  |  |  |  |  |  |  |
| 5. RAV_A_1 | 0.35 | 0.41 | 0.38 | 0.42 | 1.00 |  |  |  |  |  |  |  |  |  |  |  |  |  |  |  |
| 6. RAV_B_1 | 0.39 | 0.41 | 0.37 | 0.41 | 0.63 | 1.00 |  |  |  |  |  |  |  |  |  |  |  |  |  |  |
| 7. RAV_C_1 | 0.46 | 0.52 | 0.45 | 0.51 | 0.66 | 0.74 | 1.00 |  |  |  |  |  |  |  |  |  |  |  |  |  |
| 8. RAV_D_1 | 0.44 | 0.48 | 0.44 | 0.51 | 0.62 | 0.75 | 0.75 | 1.00 |  |  |  |  |  |  |  |  |  |  |  |  |
| 9. RAV_E_1 | 0.53 | 0.61 | 0.53 | 0.61 | 0.52 | 0.58 | 0.73 | 0.66 | 1.00 |  |  |  |  |  |  |  |  |  |  |  |
| 10. RAV_A_2 | 0.28 | 0.31 | 0.29 | 0.34 | 0.50 | 0.48 | 0.48 | 0.48 | 0.39 | 1.00 |  |  |  |  |  |  |  |  |  |  |
| 11. RAV_B_2 | 0.35 | 0.36 | 0.34 | 0.41 | 0.57 | 0.63 | 0.61 | 0.68 | 0.51 | 0.49 | 1.00 |  |  |  |  |  |  |  |  |  |
| 12. RAV_C_2 | 0.45 | 0.51 | 0.47 | 0.51 | 0.59 | 0.64 | 0.73 | 0.68 | 0.68 | 0.51 | 0.69 | 1.00 |  |  |  |  |  |  |  |  |
| 13. RAV_D_2 | 0.46 | 0.52 | 0.51 | 0.54 | 0.60 | 0.65 | 0.67 | 0.70 | 0.66 | 0.47 | 0.68 | 0.77 | 1.00 |  |  |  |  |  |  |  |
| 14. RAV_E_2 | 0.51 | 0.56 | 0.54 | 0.60 | 0.51 | 0.54 | 0.67 | 0.61 | 0.82 | 0.43 | 0.54 | 0.73 | 0.69 | 1.00 |  |  |  |  |  |  |
| 15. PROP_1 | 0.52 | 0.57 | 0.51 | 0.54 | 0.40 | 0.41 | 0.57 | 0.49 | 0.72 | 0.34 | 0.36 | 0.55 | 0.52 | 0.70 | 1.00 |  |  |  |  |  |
| 16. PROP_2 | 0.53 | 0.58 | 0.52 | 0.57 | 0.44 | 0.43 | 0.58 | 0.52 | 0.72 | 0.33 | 0.39 | 0.56 | 0.56 | 0.69 | 0.83 | 1.00 |  |  |  |  |
| 17. ARITH_1 | 0.51 | 0.53 | 0.52 | 0.59 | 0.53 | 0.54 | 0.59 | 0.59 | 0.67 | 0.36 | 0.55 | 0.60 | 0.62 | 0.67 | 0.55 | 0.59 | 1.00 |  |  |  |
| 18. ARITH_2 | 0.52 | 0.51 | 0.51 | 0.53 | 0.50 | 0.55 | 0.55 | 0.58 | 0.58 | 0.43 | 0.52 | 0.61 | 0.59 | 0.61 | 0.48 | 0.57 | 0.70 | 1.00 |  |  |
| 19. ALG_1 | 0.58 | 0.63 | 0.58 | 0.65 | 0.52 | 0.53 | 0.65 | 0.60 | 0.79 | 0.38 | 0.51 | 0.69 | 0.66 | 0.77 | 0.76 | 0.80 | 0.75 | 0.65 | 1.00 |  |
| 20. ALG_2 | 0.56 | 0.61 | 0.58 | 0.62 | 0.52 | 0.56 | 0.63 | 0.62 | 0.74 | 0.38 | 0.50 | 0.66 | 0.67 | 0.76 | 0.71 | 0.76 | 0.69 | 0.67 | 0.87 | 1.00 |
| 21. NWORDRC1 | 0.50 | 0.50 | 0.50 | 0.50 | 0.45 | 0.43 | 0.50 | 0.48 | 0.59 | 0.29 | 0.39 | 0.51 | 0.55 | 0.60 | 0.53 | 0.59 | 0.55 | 0.61 | 0.66 | 0.68 |
| 22. NWORDRC2 | 0.49 | 0.47 | 0.46 | 0.47 | 0.47 | 0.40 | 0.47 | 0.42 | 0.52 | 0.31 | 0.38 | 0.45 | 0.48 | 0.56 | 0.50 | 0.56 | 0.54 | 0.53 | 0.62 | 0.63 |
| 23. CH_CL_1 | 0.41 | 0.42 | 0.42 | 0.43 | 0.37 | 0.32 | 0.41 | 0.39 | 0.46 | 0.28 | 0.32 | 0.41 | 0.44 | 0.48 | 0.42 | 0.47 | 0.49 | 0.48 | 0.56 | 0.57 |
| 24. CH_CL_2 | 0.44 | 0.42 | 0.43 | 0.44 | 0.40 | 0.37 | 0.45 | 0.49 | 0.48 | 0.31 | 0.40 | 0.45 | 0.51 | 0.50 | 0.42 | 0.48 | 0.52 | 0.50 | 0.56 | 0.59 |
| 25. DAT_LH1 | 0.43 | 0.36 | 0.40 | 0.40 | 0.34 | 0.37 | 0.44 | 0.39 | 0.41 | 0.29 | 0.38 | 0.45 | 0.43 | 0.44 | 0.38 | 0.43 | 0.49 | 0.49 | 0.52 | 0.51 |
| 26. DAT_RH1 | 0.52 | 0.48 | 0.53 | 0.51 | 0.48 | 0.51 | 0.54 | 0.53 | 0.57 | 0.33 | 0.45 | 0.54 | 0.58 | 0.60 | 0.51 | 0.57 | 0.64 | 0.61 | 0.65 | 0.65 |
| 27. DAT_LH2 | 0.40 | 0.38 | 0.41 | 0.39 | 0.41 | 0.36 | 0.39 | 0.40 | 0.39 | 0.31 | 0.32 | 0.42 | 0.42 | 0.44 | 0.36 | 0.44 | 0.45 | 0.47 | 0.49 | 0.55 |
| 28. DAT_RH2 | 0.57 | 0.51 | 0.54 | 0.54 | 0.50 | 0.49 | 0.55 | 0.54 | 0.57 | 0.34 | 0.44 | 0.56 | 0.60 | 0.62 | 0.51 | 0.58 | 0.64 | 0.64 | 0.65 | 0.70 |
| 29. S_ATT1 | 0.28 | 0.22 | 0.25 | 0.21 | 0.26 | 0.24 | 0.26 | 0.29 | 0.28 | 0.14 | 0.27 | 0.30 | 0.30 | 0.29 | 0.23 | 0.30 | 0.31 | 0.28 | 0.32 | 0.32 |
| 30. S_ATT2 | 0.28 | 0.25 | 0.21 | 0.22 | 0.35 | 0.27 | 0.27 | 0.23 | 0.26 | 0.21 | 0.25 | 0.26 | 0.30 | 0.34 | 0.22 | 0.26 | 0.31 | 0.29 | 0.29 | 0.29 |
| 31. EXTR_CH | 0.19 | 0.22 | 0.20 | 0.14 | 0.21 | 0.13 | 0.19 | 0.18 | 0.20 | 0.20 | 0.15 | 0.20 | 0.20 | 0.18 | 0.16 | 0.18 | 0.24 | 0.18 | 0.23 | 0.24 |
| 32. AGREE_CH | 0.06 | 0.09 | 0.07 | 0.07 | 0.14 | 0.11 | 0.11 | 0.16 | 0.08 | 0.07 | 0.08 | 0.09 | 0.13 | 0.01 | −0.01 | 0.04 | 0.09 | 0.09 | 0.09 | 0.09 |
| 33. CONS_CH | −0.16 | −0.16 | −0.16 | −0.15 | −0.11 | −0.10 | −0.17 | −0.12 | −0.21 | −0.07 | −0.11 | −0.15 | −0.15 | −0.25 | −0.22 | −0.15 | −0.18 | −0.10 | −0.26 | −0.24 |
| 34. EMO_CH | 0.14 | 0.14 | 0.13 | 0.12 | 0.11 | 0.13 | 0.16 | 0.16 | 0.13 | 0.18 | 0.15 | 0.16 | 0.17 | 0.14 | 0.10 | 0.14 | 0.17 | 0.16 | 0.14 | 0.14 |
| 35. INTEL_CH | 0.05 | 0.01 | 0.04 | 0.05 | 0.10 | 0.10 | 0.12 | 0.10 | 0.09 | 0.09 | 0.08 | 0.08 | 0.08 | 0.04 | 0.08 | 0.09 | 0.10 | 0.08 | 0.10 | 0.10 |
| Mean | 6.22 | 4.55 | 6.73 | 5.00 | 10.83 | 9.67 | 8.10 | 8.30 | 4.78 | 11.16 | 10.29 | 8.78 | 8.69 | 5.69 | 22.50 | 25.65 | 5.55 | 6.19 | 6.43 | 7.39 |
| S.D. | 1.35 | 1.43 | 1.23 | 1.50 | 1.46 | 2.66 | 2.87 | 2.99 | 3.50 | 1.14 | 2.24 | 2.59 | 2.74 | 3.44 | 16.40 | 18.94 | 1.72 | 1.67 | 3.54 | 3.49 |

| Variables | 21 | 22 | 23 | 24 | 25 | 26 | 27 | 28 | 29 | 30 | 31 | 32 | 33 | 34 | 35 |
| --- | --- | --- | --- | --- | --- | --- | --- | --- | --- | --- | --- | --- | --- | --- | --- |
| 21. WORDRC1 | 1.00 |  |  |  |  |  |  |  |  |  |  |  |  |  |  |
| 22. WORDRC2 | 0.78 | 1.00 |  |  |  |  |  |  |  |  |  |  |  |  |  |
| 23. CH_CL_1 | 0.64 | 0.58 | 1.00 |  |  |  |  |  |  |  |  |  |  |  |  |
| 24. CH_CL_2 | 0.62 | 0.59 | 0.70 | 1.00 |  |  |  |  |  |  |  |  |  |  |  |
| 25. DAT_LH1 | 0.59 | 0.60 | 0.58 | 0.60 | 1.00 |  |  |  |  |  |  |  |  |  |  |
| 26. DAT_RH1 | 0.72 | 0.68 | 0.67 | 0.71 | 0.75 | 1.00 |  |  |  |  |  |  |  |  |  |
| 27. DAT_LH2 | 0.58 | 0.62 | 0.51 | 0.58 | 0.70 | 0.68 | 1.00 |  |  |  |  |  |  |  |  |
| 28. DAT_RH2 | 0.70 | 0.68 | 0.62 | 0.73 | 0.70 | 0.85 | 0.75 | 1.00 |  |  |  |  |  |  |  |
| 29. S_ATT1 | 0.29 | 0.33 | 0.32 | 0.46 | 0.36 | 0.51 | 0.34 | 0.41 | 1.00 |  |  |  |  |  |  |
| 30. S_ATT2 | 0.32 | 0.40 | 0.39 | 0.28 | 0.35 | 0.41 | 0.34 | 0.43 | 0.38 | 1.00 |  |  |  |  |  |
| 31. EXTR_CH | 0.24 | 0.24 | 0.22 | 0.23 | 0.25 | 0.31 | 0.24 | 0.28 | 0.16 | 0.13 | 1.00 |  |  |  |  |
| 32. AGREE_CH | 0.16 | 0.13 | 0.10 | 0.18 | 0.14 | 0.16 | 0.19 | 0.12 | 0.07 | 0.05 | 0.32 | 1.00 |  |  |  |
| 33. CONS_CH | −0.19 | −0.18 | −0.16 | −0.16 | −0.17 | −0.22 | −0.12 | −0.19 | −0.13 | −0.05 | −0.11 | 0.21 | 1.00 |  |  |
| 34. EMO_CH | 0.07 | 0.05 | 0.09 | 0.08 | 0.03 | 0.12 | 0.03 | 0.11 | 0.03 | 0.00 | 0.26 | 0.06 | 0.17 | 1.00 |  |
| 35. INTEL_CH | 0.07 | 0.11 | 0.06 | 0.05 | 0.07 | 0.05 | 0.09 | 0.07 | 0.00 | 0.07 | 0.31 | 0.30 | 0.25 | 0.14 | 1.00 |
| Mean | −0.55 | −0.53 | −0.65 | −0.64 | −0.73 | −1.15 | −0.72 | −1.01 | −0.19 | −0.16 | 34.01 | 37.92 | 35.71 | 32.23 | 35.46 |
| S.D. | 0.12 | 0.11 | 0.22 | 0.21 | 0.38 | 0.68 | 0.30 | 0.48 | 0.24 | 0.20 | 5.69 | 6.12 | 7.16 | 6.90 | 5.55 |

Symbols FDS and BDS stand for forward and backward digit span, respectively; symbols RAV_A-E stand for the five sets of SPM of the Raven test; symbols PROP, ARITH, and ALG stand for proportional, arithmetic, and algebraic reasoning, respectively; the symbols WORDRC and CH-CL stand for reaction times in word recognition and choice reaction tasks, respectively; the symbols DAT_LH, DAT_RH, and S_ATT stand for reaction times to divided attention and Stroop incompatible tasks; symbols EXTR_CH, AGREE_CH, CONS_CH, EMO_CH, and INTEL_CH stand for children’s self-rating scores on extroversion, agreeableness, conscientiousness, neuroticism, and openness, respectively. Z scores were used for reaction times to facilitate model estimation. Numbers indicate testing waves.

**Table S3.** Correlations, means, SD of the variables used in Study 3 (total sample).

| Variables | 21 | 22 | 23 | 24 | 25 | 26 | 27 | 28 | 29 | 30 | 31 | 32 | 33 | 34 | 35 | 36 | 37 | 38 | 39 | 40 |
| --- | --- | --- | --- | --- | --- | --- | --- | --- | --- | --- | --- | --- | --- | --- | --- | --- | --- | --- | --- | --- |
| 21. MATH1 | 1.00 |  |  |  |  |  |  |  |  |  |  |  |  |  |  |  |  |  |  |  |
| 22. SUP1 | 0.37 | 1.00 |  |  |  |  |  |  |  |  |  |  |  |  |  |  |  |  |  |  |
| 23. SUP2 | 0.32 | 0.47 | 1.00 |  |  |  |  |  |  |  |  |  |  |  |  |  |  |  |  |  |
| 24. SOC2 | 0.29 | 0.55 | 0.49 | 1.00 |  |  |  |  |  |  |  |  |  |  |  |  |  |  |  |  |
| 25. SPOR | 0.43 | 0.33 | 0.33 | 0.22 | 1.00 |  |  |  |  |  |  |  |  |  |  |  |  |  |  |  |
| 26. F2ACHIE | 0.26 | 0.20 | 0.16 | 0.22 | 0.19 | 1.00 |  |  |  |  |  |  |  |  |  |  |  |  |  |  |
| 27. F2ORG | 0.17 | 0.23 | 0.27 | 0.25 | 0.12 | 0.64 | 1.00 |  |  |  |  |  |  |  |  |  |  |  |  |  |
| 28. F1EMO | −0.03 | −0.06 | 0.04 | −0.10 | −0.06 | −0.18 | −0.12 | 1.00 |  |  |  |  |  |  |  |  |  |  |  |  |
| 29. F1EGO | 0.04 | −0.04 | 0.07 | −0.12 | 0.03 | −0.16 | −0.10 | 0.55 | 1.00 |  |  |  |  |  |  |  |  |  |  |  |
| 30. F5PROS | −0.08 | 0.01 | 0.11 | −0.03 | 0.01 | 0.14 | 0.16 | 0.20 | 0.19 | 1.00 |  |  |  |  |  |  |  |  |  |  |
| 31. F5EXT | 0.01 | 0.08 | 0.18 | 0.21 | 0.16 | 0.23 | 0.22 | 0.01 | −0.04 | 0.50 | 1.00 |  |  |  |  |  |  |  |  |  |
| 32. F4HELP | −0.07 | 0.13 | 0.28 | 0.18 | 0.01 | 0.26 | 0.40 | −0.03 | −0.07 | 0.29 | 0.25 | 1.00 |  |  |  |  |  |  |  |  |
| 33. F4AGR | −0.02 | 0.07 | 0.21 | 0.15 | 0.06 | 0.32 | 0.45 | −0.13 | −0.14 | 0.22 | 0.34 | 0.65 | 1.00 |  |  |  |  |  |  |  |
| 34. F3INTE | 0.34 | 0.33 | 0.24 | 0.21 | 0.22 | 0.39 | 0.50 | −0.07 | 0.03 | 0.16 | 0.22 | 0.19 | 0.26 | 1.00 |  |  |  |  |  |  |
| 35. F3OPEN | 0.01 | 0.12 | 0.16 | 0.10 | 0.11 | 0.15 | 0.19 | 0.21 | 0.23 | 0.26 | 0.25 | 0.10 | 0.18 | 0.42 | 1.00 |  |  |  |  |  |
| 36. SPATIAL | 0.02 | 0.14 | 0.08 | 0.16 | 0.00 | −0.08 | −0.12 | 0.05 | 0.11 | −0.03 | −0.09 | −0.12 | −0.11 | −0.05 | 0.06 | 1.00 |  |  |  |  |
| 37. QUANT | 0.07 | 0.20 | 0.08 | 0.11 | −0.01 | −0.01 | −0.03 | −0.04 | 0.03 | −0.04 | −0.01 | 0.02 | −0.03 | 0.05 | −0.01 | 0.42 | 1.00 |  |  |  |
| 38. CAUSAL | 0.00 | 0.17 | 0.06 | 0.18 | −0.09 | 0.06 | 0.05 | 0.03 | 0.09 | 0.09 | −0.05 | 0.10 | −0.07 | −0.01 | 0.10 | 0.44 | 0.43 | 1.00 |  |  |
| 39. QUAL | 0.10 | 0.09 | 0.03 | 0.08 | 0.02 | −0.03 | −0.02 | −0.04 | 0.06 | −0.06 | −0.19 | 0.09 | −0.06 | −0.04 | −0.04 | 0.29 | 0.28 | 0.32 | 1.00 |  |
| 40. SOCIAL | 0.16 | 0.32 | 0.13 | 0.22 | 0.12 | 0.07 | 0.06 | 0.08 | 0.00 | 0.01 | −0.01 | 0.00 | −0.08 | 0.06 | 0.07 | 0.29 | 0.31 | 0.26 | 0.19 | 1.00 |
| 41. TCWL | −0.07 | 0.03 | −0.01 | −0.01 | −0.12 | 0.22 | 0.19 | −0.08 | 0.03 | 0.17 | 0.13 | 0.22 | 0.14 | 0.07 | −0.03 | −0.06 | 0.01 | 0.28 | 0.03 | 0.00 |
| 42. PEGD | 0.05 | 0.16 | 0.13 | 0.23 | 0.00 | 0.13 | 0.13 | 0.02 | 0.12 | 0.21 | 0.23 | 0.29 | 0.18 | 0.11 | 0.20 | 0.24 | 0.29 | 0.32 | 0.16 | 0.12 |
| 43. COMEM | −0.05 | 0.24 | 0.08 | 0.22 | −0.08 | 0.08 | 0.04 | −0.01 | 0.02 | 0.19 | 0.03 | 0.17 | −0.02 | 0.07 | 0.08 | 0.26 | 0.34 | 0.44 | 0.26 | 0.33 |
| 44. STORY | 0.03 | 0.17 | 0.03 | 0.16 | 0.08 | 0.09 | 0.08 | −0.16 | 0.01 | 0.13 | 0.09 | 0.15 | 0.14 | 0.06 | −0.02 | 0.23 | 0.18 | 0.21 | 0.12 | 0.24 |
| 45. FAC_JOY | 0.12 | 0.29 | 0.22 | 0.13 | 0.24 | 0.21 | 0.32 | −0.20 | 0.01 | −0.01 | 0.06 | 0.12 | 0.16 | 0.18 | 0.10 | −0.02 | 0.00 | 0.14 | −0.04 | 0.11 |
| 46. FAC_GRIF | 0.01 | 0.19 | 0.09 | 0.12 | −0.01 | −0.04 | 0.01 | 0.12 | 0.00 | 0.11 | −0.02 | 0.14 | 0.07 | 0.06 | 0.05 | 0.03 | 0.00 | 0.15 | 0.09 | 0.19 |
| 47. FAC_SURP | 0.16 | 0.43 | 0.36 | 0.33 | 0.12 | 0.26 | 0.25 | 0.02 | 0.01 | 0.18 | 0.13 | 0.33 | 0.26 | 0.27 | 0.10 | 0.08 | 0.11 | 0.29 | 0.14 | 0.20 |
| 48. GREEK | 0.24 | 0.37 | 0.15 | 0.24 | 0.05 | 0.42 | 0.30 | −0.21 | −0.13 | 0.03 | 0.08 | 0.10 | 0.09 | 0.28 | 0.00 | 0.10 | 0.13 | 0.26 | 0.14 | 0.20 |
| 49. MATHS | 0.29 | 0.27 | 0.10 | 0.18 | 0.02 | 0.39 | 0.26 | −0.13 | −0.04 | 0.05 | 0.02 | 0.07 | 0.00 | 0.26 | −0.03 | 0.13 | 0.22 | 0.34 | 0.18 | 0.22 |
| Mean | 3.11 | 3.15 | 3.26 | 3.31 | 2.90 | 3.59 | 3.54 | 2.53 | 2.52 | 3.42 | 3.87 | 3.62 | 3.69 | 3.67 | 3.35 | 1.00 | 0.71 | −0.30 | 2.08 | 0.57 |
| S.D. | 0.85 | 0.75 | 0.74 | 0.71 | 0.92 | 0.98 | 0.84 | 0.93 | 0.97 | 0.84 | 0.75 | 0.90 | 0.76 | 0.75 | 0.94 | 1.55 | 1.69 | 1.57 | 1.56 | 1.84 |

| Variables | 41 | 42 | 43 | 44 | 45 | 46 | 47 | 48 | 49 |
| --- | --- | --- | --- | --- | --- | --- | --- | --- | --- |
| 41. TCWL | 1.00 |  |  |  |  |  |  |  |  |
| 42. PEGD | 0.30 | 1.00 |  |  |  |  |  |  |  |
| 43. COMEM | 0.30 | 0.39 | 1.00 |  |  |  |  |  |  |
| 44. STORY | 0.13 | 0.25 | 0.26 | 1.00 |  |  |  |  |  |
| 45. FAC_JOY | 0.19 | 0.07 | 0.06 | 0.05 | 1.00 |  |  |  |  |
| 46. FAC_GRIF | 0.33 | 0.13 | 0.27 | −0.04 | 0.25 | 1.00 |  |  |  |
| 47. FAC_SURP | 0.24 | 0.24 | 0.33 | 0.18 | 0.34 | 0.38 | 1.00 |  |  |
| 48. GREEK | 0.22 | 0.12 | 0.24 | 0.20 | 0.06 | 0.02 | 0.31 | 1.00 |  |
| 49. MATHS | 0.23 | 0.16 | 0.29 | 0.25 | 0.09 | 0.03 | 0.23 | 0.82 | 1.00 |
| Mean | 4.33 | 3.75 | 5.30 | 3.05 | −0.05 | −0.15 | −0.22 | 4.46 | 4.59 |
| S.D. | 0.75 | 0.89 | 2.21 | 1.64 | 1.02 | 1.00 | 1.06 | 1.47 | 1.49 |

SR1SELFR, SR2SELFR, and SR50SELF stand for self-representation of self-monitoring and self-regulation ability. EI17, EI18, EI20, and EI21 stand for trait emotional intelligence self-ratings in self-awareness about emotions; EI8, EI15, EI31, EI35 stand for self-ratings in emotional stability; EI13, EI19, EI25, EI26 stand for self-ratings in recognition and management of emotional signals; SEA_F1, SEA_SD1, SEA_M5, and SEA_S1 stand for self-evaluation accuracy in spatial, quantitative, Raven-like matrices, and scientific reasoning, respectively. SRMATH1, SRSUP1, SRSUP2, SRSOC2, and SRSPOR stand for self-representation scores in specific domains (mathematics, causal, social, verbal, respectively); symbols starting with F (F1-F5) stand Big Five Facets in achievement (F2ACHIE) and order (f2ORG) for C, anxiety (F1EMO) and self-consciousness (F1EGO) for N ; extroversion (F5EXT) and introversion (F5PROS )for E; altruism (F4HELP) , and agreeableness F4AGR) for A; intellect (F3INTE) and openness F3OPEN) for O; The symbols SPATIAL, QUANT, CAUSAL, INDUCT, SOCIAL, stand for spatial, quantitative, causal, inductive, and social reasoning, respectively; the symbols PEGD, COMEM, and STORY stand for performance scores on association of different emotions with corresponding real-life situation and FAC_JOY FAC_GRIF FAC_SURP stand for the specification of characteristics of different emotions; GREEK and MATHS stand for mean performance in Greek and mathematics as scored by teachers.
