# Supplementary material for: Mind-Personality Relations from Childhood to Early Adulthood
Source: J Intell. 2018 Dec 6;6(4):51. doi: 10.3390/jintelligence6040051 (PMC6480795; doi:10.3390/jintelligence6040051)
Supplement: Supplementary file 1 [file jintelligence-06-00051-s001.zip › Supplementary_2_Codes for SEM models.docx]

**SUPPLEMENTARY FILE PRESENTING MODEL CODES AND FULL STANDARDIZED SOLUTIONS FOR THE MAIN MODELS PRESENTED IN THE PAPER.**

All Confirmatory Factor Analysis and Structural Equation Models were tested by EQS 6.1. Latent transition models were tested by Mplus 7.31. Thus, the model codes below must be read in conjunction with the language specification of each of these two programs.

The correlations and statistics of the variables used in the various models are presented in Supplementary Tables. Below only variables used in the model concerned are mentioned.

**STUDY 1**

**Model 1: Model for specifying the correlation between g and the GFP at first testing wave.**

/VARIABLES

V4=MATRICES1; V7=DEDUCTIVE1; V13=SCIENTIFIC1;

V19=P1; V20=N1; V21=E1; V22=L1;

F1=GF1; F4=GFP1; F7=P; F8=N; F9=E; F10=L;

/EQUATIONS

V4= 1F1+E4;

V7= *F1+E7;

V13= *F1+E13;

V27= F4+E27;

V28= *F4+E28;

V29= *F4+E29;

V83= *F4+E83;

/VAR

F1=*;

F4=*;

All error variances were free to be estimated.

/COV

F1,F4=*;

/END

STANDARDIZED SOLUTION: R-SQUARED

MAT1 =V4 = .752 F1 + .659 E4 .566

DED1 =V7 = .615*F1 + .788 E7 .378

SCI1 =V13 = .723*F1 + .691 E13 .522

P1 =V27 = .494 F4 + .870 E27 .244

N1 =V28 = .528*F4 + .849 E28 .279

E1 =V29 = -.002*F4 +1.000 E29 .000

L1I =V83 = 1.000*F4 + .000 E83 1.000

CORRELATIONS AMONG INDEPENDENT VARIABLES

I F4 - GFP1 .590*I

I F1 - GF1 I

I I

GOODNESS OF FIT SUMMARY FOR METHOD = ML

MODEL AIC = 12.540 MODEL CAIC = -46.749

CHI-SQUARE = 38.540 BASED ON 13 DEGREES OF FREEDOM

PROBABILITY VALUE FOR THE CHI-SQUARE STATISTIC IS .00024

COMPARATIVE FIT INDEX (CFI) = .940

STANDARDIZED RMR = .062

ROOT MEAN-SQUARE ERROR OF APPROXIMATION (RMSEA) = .087

90% CONFIDENCE INTERVAL OF RMSEA ( .056, .119)

RELIABILITY COEFFICIENTS

------------------------

CRONBACH'S ALPHA = .668

RELIABILITY COEFFICIENT RHO = .777

**Model 2: Growth model for Gf at the three testing waves; personality factors at first wave were used as factors influencing the intercept and slope of Gf growth factors.**

/VARIABLES

V77=MGF_Z1; V78=MGF_Z2; V79=MGF_Z3;

V27=Z_P1; V28=Z_N1; V29=Z_E1; V30=Z_L1;

F1=INTER_GF;

F2=SLOPE_GF;

F9=E;

F10=L

F11=P;

F12=N;

/EQUATIONS

V77= 1F1+0F2+E77;

V78= 1F1+1F2+E78;

V79= 1F1+2F2+E79;

V27= F11+E27;

V28= F12+E28;

V29= F9+E29;

V30= F10+E30;

F1=0*V999+*d9+*d11+*d12+*f10+D1;

F2=0*V999+*d9+*d11+*d12+*f10+D2;

F11=*F10+D11;

F12=*F10+D12;

F9= *F10+D9;

/VAR

V999=1;

F10=*;

E77 TO E79=*;

E27 to E28 =.2;

D1 TO D2=*;

D9=*;

D11 to D12=*;

/COV

E29,E27=*;

/END

STANDARDIZED SOLUTION: R-SQUARED

Z_P1 =V27 = .885 F11 + .465 E27 .784

Z_N1 =V28 = .894 F12 + .447 E28 .800

Z_E1 =V29 = .895 F9 + .445 E29 .802

Z_L1 =V30 = .895 F10 + .447 E30 .801

MGF_Z1 =V77 = .887 F1 + .461 E77 .787

MGF_Z2 =V78 = .899 F1 + .293 F2 + .509 E78 .741

MGF_Z3 =V79 = .956 F1 + .624 F2 + .212 E79 .955

INTER_GF=F1 = .000*V999 - .678*F10 + .640 D1 - .036*D9

- .288*D11 - .216*D12 .591

SLOPE_GF=F2 = .000*V999 + .266*F10 + .900 D2 + .185*D9

+ .244*D11 + .163*D12 .190

E =F9 = .029*F10 +1.000 D9 .001

P =F11 = -.598*F10 + .802 D11 .357

N =F12 = -.643*F10 + .766 D12 .414

GOODNESS OF FIT SUMMARY FOR METHOD = ML

MODEL AIC = -7.286 MODEL CAIC = -61.921

CHI-SQUARE = 16.714 BASED ON 12 DEGREES OF FREEDOM

PROBABILITY VALUE FOR THE CHI-SQUARE STATISTIC IS .16067

FIT INDICES (BASED ON COVARIANCE MATRIX ONLY, NOT THE MEANS)

COMPARATIVE FIT INDEX (CFI) = .997

ROOT MEAN-SQUARE ERROR OF APPROXIMATION (RMSEA) = .073

90% CONFIDENCE INTERVAL OF RMSEA ( .026, .119)

SATORRA-BENTLER SCALED CHI-SQUARE = 16.2722 ON 12 DEGREES OF FREEDOM

PROBABILITY VALUE FOR THE CHI-SQUARE STATISTIC IS .17908

COMPARATIVE FIT INDEX (CFI) = .965

ROOT MEAN-SQUARE ERROR OF APPROXIMATION (RMSEA) = .037

90% CONFIDENCE INTERVAL OF RMSEA ( .000, .078)

**Latent Transition Analysis**

**Model 3: Personality Influences on Cognitive Transition**

CATEGORICAL = Mat1 Mat2 Prop1 Prop2 Form2_1 Form2_2;

USEVARIABLES = Mat1 Mat2 Prop1 Prop2 Form2_1 Form2_2

P1 N1 E1 L1;

CLASSES = c1 (2) c2 (2);

ANALYSIS: TYPE = MIXTURE;

STARTS = 20 10;

MODEL: %OVERALL%

[c2#1] (a);

c2 ON c1 P1 E1 N1 L1 (b);

c1 ON P1 E1 N1 L1;

MODEL c1: %c1#1%

[Mat1$1 Prop1$1*-3 Scien_1$1] (1-3);

%c1#2%

[Mat1$1 Prop1$1*-3 Scien_1$1] (4-6);

MODEL c2: %c2#1%

[Mat2$1*-5 Prop2$1 Scien_2$1] (1-3);

%c2#2%

[Mat2$1*-5 Prop2$1 Scien_2$1] (4-6);

**Model 4: Cognitive Influences on Personality Transition**

CATEGORICAL = P1L N1L L1L P2L N2L L2L;

USEVARIABLES = P1L N1L L1L P2L N2L L2L

GF;

CLASSES = c1 (2) c2 (2);

DEFINE:

GF = (Mat1 + Prop1 + Form2_1)/3;

ANALYSIS: TYPE = MIXTURE;

STARTS = 100 20;

MODEL: %OVERALL%

[c2#1] (a);

c2 ON c1 (b);

c1 ON GF;

MODEL c1: %c1#1%

[P1L$1 N1L$1 L1L$1];

c2 ON GF;

%c1#2%

[P1L$1 N1L$1 L1L$1];

c2 ON GF;

MODEL c2: %c2#1%

[P2L$1 N2L$1 L2L$1];

%c2#2%

[P2L$1 N2L$1 L2L$1];

**STUDY 2**

**Model 1: Model specifying the correlation between Gf at second testing wave and GFP**

VARIABLES

F1=Gf2;

F2=GFP;

F20=SPEED2;

F21=ATCON2;

F22=WM2;

F23=RAV2;

F24=MATH2;

F151=STA-A;

F152=PLA-B;

/EQUATIONS

E = F152 + E268;

A = F151 + E269;

C = *F151 + E270;

N = *F151 + E271;

O = *F152 + E272;

Sp1_2 = *F20 + E64;

Sp2_2 = F20 + E62;

DAT1_2 = F21 + E77;

DAT2_2 = *F21 + E78;

SAT1_2 = *F21 + E80;

FDS_2 = *F22 + E29;

BDS_2 = F22 + E30;

RAV_A2 = *F23 + E47;

RAV_B2 = F23 + E48;

RAV_C2 = *F23 + E49;

RAV_D2 = *F23 + E50;

RAV_E2 = *F23 + E51;

ARI_2 = *F24 + E54;

PRO_2 = F24 + E56;

ALG_2 = *F24 + E57;

F20 = F1 + D20;

F21 = *F1 + D21;

F22 = *F1 + D22;

F23 = *F1 + D23;

F24 = *F1 + D24;

F151= *F2 + D151;

F152= F2 + D152;

/VARIANCES

F1 TO F2=*;

All error variances and factor disturbances were free to be estimated.

/COVARIANCES

F1,F2=*;

STANDARDIZED SOLUTION FOR THE MODEL ABOVE: R-SQUARED

FDS_2 =V29 = .810*F22 + .586 E29 .657

BDS_2 =V30 = .851 F22 + .526 E30 .724

RAV_A_2 =V47 = .557*F23 + .831 E47 .310

RAV_B_2 =V48 = .736 F23 + .677 E48 .542

RAV_C_2 =V49 = .882*F23 + .471 E49 .778

RAV_D_2 =V50 = .861*F23 + .508 E50 .742

RAV_E_2 =V51 = .829*F23 + .559 E51 .687

PROP_2 =V54 = .801*F24 + .598 E54 .642

ARITH_2 =V56 = .757 F24 + .654 E56 .573

ALG_2 =V58 = .915*F24 + .403 E57 .837

SP1_2=V62 = .779 F20 + .627 E62 .607

SP2_2=V64 = .753*F20 + .658 E64 .567

DAT_LH2=V77 = .770 F21 + .638 E77 .594

DAT_RH2=V78 = .980*F21 + .199 E78 .960

S_ATT2 =V80 = .447*F21 + .895 E80 .200

E =V268 = .655 F152 + .755 E268 .429

A =V269 = .484 F151 + .875 E269 .235

C =V270 = .090*F151 + .996 E270 .008

N =V271 = .329*F151 + .944 E271 .108

O =V272 = .495*F152 + .869 E272 .245

SPEED2 =F20 = .949 F1 + .316 D20 .900

SELATT2 =F21 = .856*F1 + .517 D21 .733

WM2 =F22 = .809*F1 + .588 D22 .654

RAV2 =F23 = .847*F1 + .532 D23 .717

MATH2 =F24 = .953*F1 + .302 D24 .909

STA-A =F151= 1.000*F2 + .000 D151 1.000

PLA-B =F152= 1.000 F2 + .000 D152 1.000

CORRELATIONS AMONG INDEPENDENT VARIABLES

---------------------------------------

V F

--- ---

I F2 - Gf2 .347*I

I F1 - GFP I

I I

GOODNESS OF FIT SUMMARY FOR METHOD = ML

MODEL AIC = 278.280 MODEL CAIC = -539.221

CHI-SQUARE = 600.280 BASED ON 161 DEGREES OF FREEDOM

PROBABILITY VALUE FOR THE CHI-SQUARE STATISTIC IS .00000

COMPARATIVE FIT INDEX (CFI) = .908

ROOT MEAN-SQUARE ERROR OF APPROXIMATION (RMSEA) = .079

90% CONFIDENCE INTERVAL OF RMSEA ( .072, .086)

RELIABILITY COEFFICIENTS

------------------------

CRONBACH'S ALPHA = .651

RELIABILITY COEFFICIENT RHO = .816

**Model 2: Mediation of α- and β- personality factors, standing for stability and plasticity, between processing and representational efficiency and first and second testing**

VARIABLES

F10=SPEED1;

F11=SELATT1;

F20=speed2;

F21=SELATT2;

F12=WM1;

F22=WM2;

F13=RAV1;

F14=MATH1;

F23=RAV2;

F24=MATH2;

F151=STA-A;

F152=PLA-B;

F100=PrEf1;

F200=RIP1;

F300=PrEf2;

F400=RIP2;

/EQUATIONS

V63 = *F10 + E63;

V61 = F10 + E61;

V75 = *F11 + E75;

V76 = F11 + E76;

V79 = *F11 + E79;

V27 = *F12 + E27;

V28 = F12 + E28;

V41 = *F13 + E41;

V42 = F13 + E42;

V43 = *F13 + E43;

V44 = *F13 + E44;

V45 = *F13 + E45;

V53 = *F14 + E53;

V55 = F14 + E55;

V57 = *F14 + E57;

V268 = F152 + E268;

V269 = F151 + E269;

V270 = *F151 + E270;

V271 = *F151 + E271;

V272 = *F152 + E272;

V64 = *F20 + E64;

V62 = F20 + E62;

V77 = *F21 + E77;

V78 = F21 + E78;

V80 = *F21 + E80;

V29 = *F22 + E29;

V30 = F22 + E30;

V47 = *F23 + E47;

V48 = F23 + E48;

V49 = *F23 + E49;

V50 = *F23 + E50;

V51 = *F23 + E51;

V54 = *F24 + E54;

V56 = F24 + E56;

V58 = *F24 + E57;

F10 = *F100 + D10;

F11 = F100 + D11;

F12 = *F200 + D12;

F13 = *F200 + D13;

F14 = F200 + D14;

F20 = F300+ D20;

F21 = *F300+ D21;

F22 = *F400 + D22;

F23 = F400 + D23;

F24 = *F400 + D24;

F151= *F100 + *F200 + D151;

F152= *F100 + *F200 + D152;

F300= *F151 + *F152 + D300;

F400= *F151 + 0F152 + D400;

STANDARDIZED SOLUTION: R-SQUARED

FDS_1 =V27 = .760*F12 + .650 E27 .578

BDS_1 =V28 = .836 F12 + .549 E28 .698

FDS_2 =V29 = .791*F22 + .612 E29 .626

BDS_2 =V30 = .812 F22 + .584 E30 .659

RAV_A_1 =V41 = .719*F13 + .695 E41 .517

RAV_B_1 =V42 = .770 F13 + .638 E42 .593

RAV_C_1 =V43 = .877*F13 + .481 E43 .768

RAV_D_1 =V44 = .849*F13 + .528 E44 .721

RAV_E_1 =V45 = .822*F13 + .569 E45 .676

RAV_A_2 =V47 = .553*F23 + .833 E47 .305

RAV_B_2 =V48 = .768 F23 + .640 E48 .590

RAV_C_2 =V49 = .882*F23 + .472 E49 .777

RAV_D_2 =V50 = .859*F23 + .512 E50 .738

RAV_E_2 =V51 = .808*F23 + .590 E51 .652

PROP_1 =V53 = .733*F14 + .680 E53 .538

PROP_2 =V54 = .697*F24 + .717 E54 .486

ARITH_1 =V55 = .756 F14 + .655 E55 .571

ARITH_2 =V56 = .755 F24 + .656 E56 .569

ALG_1 =V57 = .950*F14 + .313 E57 .902

ALG_2 =V58 = .947*F24 + .321 E57 .897

SP1_1 =V61 = .811 F10 + .585 E61 .658

SP2_2 =V62 = .812 F20 + .584 E62 .659

SP2_1 =V63 = .739*F10 + .674 E63 .546

SP2_2 =V64 = .791*F20 + .611 E64 .626

DAT_LH1=V75 = .791*F11 + .612 E75 .625

DAT_RH1=V76 = .968 F11 + .250 E76 .937

DAT_LH2=V77 = .759*F21 + .651 E77 .576

DAT_RH2=V78 = .969 F21 + .246 E78 .939

S_ATT1 =V79 = .512*F11 + .859 E79 .262

S_ATT2 =V80 = .439*F21 + .898 E80 .193

E =V268 = .320 F152 + .947 E268 .103

A =V269 = .134 F151 + .991 E269 .018

C =V270 = -.232*F151 + .973 E270 .054

N =V271 = .180*F151 + .984 E271 .033

O =V272 = .084*F152 + .996 E272 .007

SPEED1 =F10 = .984*F100 + .179 D10 .968

SELATT1 =F11 = .956 F100 + .294 D11 .914

WM1 =F12 = .858*F200 + .514 D12 .736

RAV1 =F13 = .907*F200 + .421 D13 .823

MATH1 =F14 = .931 F200 + .364 D14 .868

SPEED2 =F20 = .948 F300 + .320 D20 .898

SELATT2 =F21 = .981*F300 + .196 D21 .962

WM2 =F22 = .864*F400 + .503 D22 .747

RAV2 =F23 = .918 F400 + .397 D23 .843

MATH2 =F24 = .934*F400 + .358 D24 .872

STA-A =F151= .074*F100 + .939*F200 + .039 D151 .999

PLA-B =F152= .929*F100 + .087*F200 + .017 D152 1.000

PREF2 =F300= -.022*F151 + .960*F152 + .337 D300 .886

RIP2 =F400= 1.000*F151 + .006 D400 1.000

CORRELATIONS AMONG INDEPENDENT VARIABLES

---------------------------------------

V F

--- ---

I F200- RIP1 .800*I

I F100-PREF1 I

I I

GOODNESS OF FIT SUMMARY FOR METHOD = ML

MODEL AIC = 751.868 MODEL CAIC = -2009.120

CHI-SQUARE = 1839.868 BASED ON 544 DEGREES OF FREEDOM

PROBABILITY VALUE FOR THE CHI-SQUARE STATISTIC IS .00000

COMPARATIVE FIT INDEX (CFI) = .891

ROOT MEAN-SQUARE ERROR OF APPROXIMATION (RMSEA) = .074

90% CONFIDENCE INTERVAL OF RMSEA ( .070, .078)

RELIABILITY COEFFICIENTS

------------------------

CRONBACH'S ALPHA = .826

RELIABILITY COEFFICIENT RHO = .845

**Model 3: Mediation the Big Five personality factors, standing for stability and plasticity, between processing and representational efficiency and first and second testing**

STANDARDIZED SOLUTION: R-SQUARED

FDS_1 =V27 = .765*F12 + .644 E27 .585

BDS_1 =V28 = .825 F12 + .565 E28 .680

FDS_2 =V29 = .803*F22 + .597 E29 .644

BDS_2 =V30 = .811 F22 + .585 E30 .658

RAV_A_1 =V41 = .724*F13 + .690 E41 .524

RAV_B_1 =V42 = .782 F13 + .623 E42 .611

RAV_C_1 =V43 = .882*F13 + .471 E43 .778

RAV_D_1 =V44 = .854*F13 + .521 E44 .729

RAV_E_1 =V45 = .795*F13 + .607 E45 .631

RAV_A_2 =V47 = .567*F23 + .824 E47 .321

RAV_B_2 =V48 = .787 F23 + .617 E48 .619

RAV_C_2 =V49 = .889*F23 + .457 E49 .791

RAV_D_2 =V50 = .869*F23 + .494 E50 .756

RAV_E_2 =V51 = .787*F23 + .616 E51 .620

PROP_1 =V53 = .711*F14 + .703 E53 .506

PROP_2 =V54 = .761*F24 + .649 E54 .579

ARITH_1 =V55 = .806 F14 + .592 E55 .649

ARITH_2 =V56 = .790 F24 + .613 E56 .624

ALG_1 =V57 = .899*F14 + .438 E57 .809

ALG_1 =V58 = .882*F24 + .472 E58 .778

SP1_1 =V61 = .804 F10 + .594 E61 .647

SP2_2 =V62 = .798 F20 + .602 E62 .638

SP2_1 =V63 = .724*F10 + .689 E63 .525

SP2_2 =V64 = .769*F20 + .639 E64 .592

DAT_LH1 =V75 = .780*F11 + .626 E75 .608

DAT_RH1 =V76 = .969 F11 + .246 E76 .940

DAT_LH2 =V77 = .755*F21 + .656 E77 .570

DAT_RH2 =V78 = .978 F21 + .210 E78 .956

S_ATT1 =V79 = .513*F11 + .858 E79 .263

S_ATT2 =V80 = .446*F21 + .895 E80 .199

E =V268= .999 F701 + .047 E268 .998

A =V269= .999 F702 + .049 E269 .998

C =V270= .999 F703 + .044 E270 .998

N =V271= .999 F704 + .044 E271 .998

O =V272= .998 F705 + .057 E272 .997

F10 =F10 = .934*F100 + .358 D10 .872

F11 =F11 = .835 F100 + .551 D11 .697

F12 =F12 = .847*F200 + .532 D12 .717

F13 =F13 = .837*F200 + .547 D13 .701

F14 =F14 = .962 F200 + .274 D14 .925

F20 =F20 = .939 F300 + .343 D20 .882

F21 =F21 = .935*F300 + .353 D21 .875

F22 =F22 = .856*F400 + .517 D22 .732

F23 =F23 = .875 F400 + .485 D23 .765

F24 =F24 = 1.000*F400 + .023 D24 .999

RIP1 =F200= 1.000*F100 + .024 D200 .999

PREF2 =F300= .366 D300 - .296*D701 + .366*D702

- .256*D703 + .264*D704 + .714*D705 .866

RIP2 =F400= .160 D400 - .381*D701 + .333*D702

- .264*D703 + .351*D704 + .725*D705 .974

E =F701= .527*F100 + .212*D200 + .823 D701 .322

A =F702= -.169*F100 + .322*D200 + .932 D702 .132

C =F703= -.023*F100 + .243*D200 + .970 D703 .059

N =F704= -.139*F100 + .161*D200 + .977 D704 .045

O =F705= -.058*F100 + .977*D200 + .207 D705 .957

GOODNESS OF FIT SUMMARY FOR METHOD = ML

CHI-SQUARE = 3657.281 BASED ON 568 DEGREES OF FREEDOM

PROBABILITY VALUE FOR THE CHI-SQUARE STATISTIC IS .00000

FIT INDICES

COMPARATIVE FIT INDEX (CFI) = .996

ROOT MEAN-SQUARE ERROR OF APPROXIMATION (RMSEA) = .112

90% CONFIDENCE INTERVAL OF RMSEA ( .108, .115)

RELIABILITY COEFFICIENTS

------------------------

CRONBACH'S ALPHA = .837

**Latent transition analysis examining the effect of personality factors in cognitive transition across the two testing waves.**

**Model 4: Personality influencing cognitive transition**

CATEGORICAL = ar_cl1 ar_cl2 alg_cl1 alg_cl2 pro_cl1 pro_cl2 rav_cl1 rav_cl2;

USEVARIABLES = ar_cl1 ar_cl2

alg_cl1 alg_cl2

pro_cl1 pro_cl2

rav_cl1 rav_cl2

nstrinc1 bds_1

A_PERS B_PERS;

CLASSES = c1 (2) c2 (2);

ANALYSIS: TYPE = MIXTURE;

STARTS = 100 50;

MODEL: %OVERALL%

[c2#1] (a);

c2 ON c1 (b);

c1 ON nstrinc1 bds_1 A_PERS B_PERS;

MODEL c1: %c1#1%

[ar_cl1$1 alg_cl1$1 pro_cl1$1 rav_cl1$1] (1-4);

c2 ON nstrinc1 bds_1 A_PERS B_PERS;

%c1#2%

[ar_cl1$1 alg_cl1$1 pro_cl1$1 rav_cl1$1] (5-8);

c2 ON nstrinc1 bds_1 A_PERS B_PERS;

MODEL c2: %c2#1%

[ar_cl2$1 alg_cl2$1 pro_cl2$1 rav_cl2$1] (1-4);

%c2#2%

[ar_cl2$1 alg_cl2$1 pro_cl2$1 rav_cl2$1] (5-8);

**STUDY 3**

**Model 1: The model specifying relations between Gf, GFP, and EI after partialing out the effect of likeability.**

VARIABLES

V347=LIKEABILITY;

F1=COGN;

F2=GFP;

F3=G;

F4=SCH;

F11=GF;

F12=COGN_SSS;

F13=COGN_S;

F14=SEA;

F41=GR;

F42=MATH;

F43=SCI;

F21=N;

F22=C;

F23=O;

F24=A;

F25=E;

F31=T_EI_SK-C;

F32=T_EI_STA;

F33=T_EI_SENS;

F34=A_EI_REC;

F35=A_EI_DEF;

F211=A_STA;

F212=B_PLA;

F311=EIt;

F312=EIa;

/EQU

V338= F11+E338;

V339=*F11+E339;

V340=*F11+E340;

V341=*F11+E341;

V342=*F11+E342;

V297= *V397+ F12+E297;

V299= *V397+ *F12+E299;

V300= *V397+ *F12+E300;

V302= *V397+ *F12+E302;

V305= *V397+ *F12+E305;

V41= *V397+ F13+E41;

V42= *V397+ *F13+E42;

V90= *V397+ *F13+E90;

V279= *V397+ *F14+E279;

V280= *V397+ F14+E280;

V282= *V397+ *F14+E282;

V283= *V397+ *F14+E283;

V310= *V397+ F22+E310;

V311= *V397+ *F22+E311;

V312= *V397+ F21+E312;

V313= *V397+ *F21+E313;

V314= *V397+ F25+E314;

V316= *V397+ *F25+E316;

V317= *V397+ F24+E317;

V318= *V397+ *F24+E318;

V320= *V397+ F23+E320;

V321= *V397+ *F23+E321;

V196= *V397+ F31+E196;

V197= *V397+ *F31+E197;

V199= *V397+ *F31+E199;

V200= *V397+ *F31+E200;

V194= *V397+ F32+E194;

V187= *V397+ *F32+E187;

V210= *V397+ *F32+E210;

V214= *V397+ *F32+E214;

V192= *V397+ F33+E192;

V198= *V397+ *F33+E198;

V204= *V397+ *F33+E204;

V205= *V397+ *F33+E205;

V369= F34+E369;

V370= *F34+E370;

V371= *F34+E371;

V372= *F34+E372;

V373= F35+E373;

V374= *F35+E374;

V375= *F35+E375;

F11 = *F3+D11;

F12 = F1+D12;

F13 =*F1+D13;

F14 =*F1+D14;

F21 =*F211+D21;

F22 =*F211+D22;

F23 =*F212+D23;

F24 = F211+D24;

F25 = F212+D25;

F31 =*F311+D31;

F32 =*F311+D32;

F33 = F311+D33;

F34 =*F312+D34;

F35 = F312+D35;

F211=*F2+D211;

F212= F2+D212;

F311=*F2+D311;

F312= F3+D312;

/VAR

F1 TO F3=*;

All error variances and factor disturbances were free to be estimated.

/COV

F1 TO F3=*;

/END

STANDARDIZED SOLUTION: R-SQUARED

SR1SELFR=V41 = .414 F13 + .334*V397 + .847 E41 .283

SR2SELFR=V42 = .557*F13 + .277*V397 + .783 E42 .387

SR50SELF=V90 = .565*F13 + .284*V397 + .775 E90 .400

EI8 =V187= .598*F32 + .071*V397 + .799 E187 .362

EI13 =V192= .633 F33 + .135*V397 + .762 E192 .419

EI15 =V194= .762 F32 - .003*V397 + .648 E194 .580

EI17 =V196= .573 F31 + .058*V397 + .817 E196 .332

EI18 =V197= .623*F31 + .136*V397 + .770 E197 .407

EI19 =V198= .650*F33 + .195*V397 + .734 E198 .461

EI20 =V199= .613*F31 + .069*V397 + .787 E199 .381

EI21 =V200= .486*F31 + .203*V397 + .850 E200 .277

EI25 =V204= .712*F33 + .180*V397 + .678 E204 .540

EI26 =V205= .547*F33 + .355*V397 + .758 E205 .426

EI31 =V210= .584*F32 + .073*V397 + .808 E210 .346

EI35 =V214= .509*F32 + .095*V397 + .855 E214 .269

SEA_F1 =V279= .361*F14 - .050*V397 + .931 E279 .133

SEA_SD1 =V280= .462 F14 - .178*V397 + .869 E280 .245

SEA_MT5 =V282= .339*F14 - .170*V397 + .925 E282 .144

SEA_S1 =V283= .279*F14 - .080*V397 + .957 E283 .084

SRMATH1 =V297= .304 F12 + .387*V397 + .871 E297 .242

SRSUP1 =V299= .771*F12 + .219*V397 + .598 E299 .642

SRSUP2 =V300= .683*F12 + .200*V397 + .702 E300 .507

SRSOC2 =V302= .716*F12 + .226*V397 + .661 E302 .563

SRSPOR =V305= .301*F12 + .466*V397 + .832 E305 .307

F2ACHIE =V310= .645 F22 + .234*V397 + .727 E310 .471

F2ORG =V311= .953*F22 + .179*V397 + .246 E311 .939

F1EMO =V312= .485 F21 - .065*V397 + .872 E312 .239

F1EGO =V313= .998*F21 - .063*V397 + .000 E313 1.000

F5PROS =V314= .704 F25 + .068*V397 + .707 E314 .500

F5EXT =V316= .724*F25 + .204*V397 + .659 E316 .566

F4HELP =V317= .719 F24 + .108*V397 + .687 E317 .528

F4AGR =V318= .898*F24 + .083*V397 + .431 E318 .814

F3INTE =V320= .925 F23 + .378*V397 + .042 E320 .998

F3OPEN =V321= .476*F23 + .186*V397 + .859 E321 .262

SPATIAL =V338= .604 F11 + .797 E338 .365

QUANT =V339= .644*F11 + .765 E339 .415

CAUSAL =V340= .791*F11 + .612 E340 .625

QUAL =V341= .601*F11 + .799 E341 .362

SOCIAL =V342= .601*F11 + .799 E342 .361

TCWL =V369= .441 F34 + .898 E369 .194

PEGD =V370= .594*F34 + .804 E370 .353

COMEM =V371= .773*F34 + .635 E371 .597

STORY =V372= .523*F34 + .852 E372 .274

FAC_JOY =V373= .444 F35 + .896 E373 .197

FAC_GRIF=V374= .558*F35 + .830 E374 .311

FAC_SURP=V375= .823*F35 + .568 E375 .678

GF =F11 = .833*F3 + .554 D11 .693

COGN_SSS=F12 = .994 F1 + .112 D12 .987

COGN_S =F13 = .364*F1 + .931 D13 .133

SEA =F14 = .808*F1 + .589 D14 .654

N =F21 = .027*F211 + 1.000 D21 .001

C =F22 = .667*F211 + .745 D22 .445

O =F23 = .589*F212 + .808 D23 .347

A =F24 = .677 F211 + .736 D24 .459

E =F25 = .509 F212 + .861 D25 .259

T_EI_SR =F31 = .870*F311 + .493 D31 .757

T_EI_STA=F32 = -.240*F311 + .971 D32 .057

T_EI_Esis=F33= .706 F311 + .709 D33 .498

A_EI_Spe=F34 = .934*F312 + .358 D34 .872

A_EI_Det=F35 = .678 F312 + .735 D35 .460

A_STA =F211= .920*F2 + .393 D211 .846

B_PLA =F212= .995 F2 + .097 D212 .991

EIt =F311= .671*F2 + .741 D311 .451

EIa =F312= .994 F3 + .105 D312 .989

CORRELATIONS AMONG INDEPENDENT VARIABLES

---------------------------------------

V F

--- ---

I F2 - GFP .620*I

I F1 - E1 I

I I

I F3 - G .665*I

I F1 - E1 I

I I

I F3 - G .420*I

I F2 - GFP I

I I

GOODNESS OF FIT SUMMARY FOR METHOD = ML

MODEL AIC = 38.106 MODEL CAIC = -4602.386

CHI-SQUARE = 2098.106 BASED ON 1030 DEGREES OF FREEDOM

PROBABILITY VALUE FOR THE CHI-SQUARE STATISTIC IS .00000

COMPARATIVE FIT INDEX (CFI) = .992

ROOT MEAN-SQUARE ERROR OF APPROXIMATION (RMSEA) = .065

90% CONFIDENCE INTERVAL OF RMSEA ( .061, .069)

RELIABILITY COEFFICIENTS

------------------------

CRONBACH'S ALPHA = .815

**Model 2: Bottom-up mediation of cognizance between cognitive and personality factors.**

STANDARDIZED SOLUTION: R-SQUARED

SR1SELFR=V41 = .234 F13 + .972 E41 .055

SR2SELFR=V42 = .392*F13 + .920 E42 .154

SR50SELF=V90 = .999*F13 + .034 E90 .999

EI8 =V187= .620*F32 + .785 E187 .384

EI13 =V192= .657 F33 + .753 E192 .432

EI15 =V194= .747 F32 + .665 E194 .557

EI17 =V196= .614 F31 + .790 E196 .377

EI18 =V197= .622*F31 + .783 E197 .387

EI19 =V198= .650*F33 + .760 E198 .423

EI20 =V199= .645*F31 + .764 E199 .416

EI21 =V200= .480*F31 + .877 E200 .231

EI25 =V204= .778*F33 + .628 E204 .605

EI26 =V205= .606*F33 + .796 E205 .367

EI31 =V210= .574*F32 + .819 E210 .330

EI35 =V214= .500*F32 + .866 E214 .250

HAI_F1 =V279= .407 F14 + .914 E279 .165

HAI_SD1 =V280= .521*F14 + .853 E280 .272

HAI_MT5 =V282= .352*F14 + .936 E282 .124

HAI_S1 =V283= .281*F14 + .960 E283 .079

BOTTLE =V285= .738 F1 + .674 E285 .545

CAR =V286= .508*F1 + .861 E286 .258

NA =V288= .535 F2 + .845 E288 .287

NS =V289= .691*F2 + .723 E289 .477

SEEDS =V290= .660 F3 + .751 E290 .435

COMB =V291= .566*F3 + .824 E291 .321

MAT_1 =V292= .439 F4 + .899 E292 .193

MAT_2 =V293= .747*F4 + .665 E293 .557

SOC_A =V295= .812 F5 + .583 E295 .660

SOC_B =V296= .687*F5 + .727 E296 .472

MATH1 =V297= .384 F12 + .923 E297 .147

SUP1 =V299= .784*F12 + .620 E299 .615

SUP2 =V300= .726*F12 + .688 E300 .527

SOC2 =V302= .731*F12 + .682 E302 .535

SPOR =V305= .413*F12 + .911 E305 .171

F2ACHIE =V310= .716 F22 + .698 E310 .513

F2ORG =V311= .932*F22 + .363 E311 .868

F1EMO =V312= .999 F21 + .036 E312 .999

F1EGO =V313= .484*F21 + .875 E313 .235

F5PROS =V314= .689 F25 + .724 E314 .475

F5EXT =V316= .751*F25 + .660 E316 .564

F4HELP =V317= .729 F24 + .685 E317 .531

F4AGR =V318= .877*F24 + .480 E318 .769

F3INTE =V320= .823 F23 + .568 E320 .677

F3OPEN =V321= .583*F23 + .813 E321 .339

TCWL =V369= .376 F34 + .926 E369 .142

PEGD =V370= .596*F34 + .803 E370 .356

COMEM =V371= .788*F34 + .616 E371 .621

STORY =V372= .530*F34 + .848 E372 .280

FAC_JOY =V373= .440 F35 + .898 E373 .194

FAC_GRIF=V374= .521*F35 + .854 E374 .271

FAC_SURP=V375= .885*F35 + .466 E375 .783

SPAC =F1 = .572*F10 + .820 D1 .327

QUA =F2 = .964*F10 + .267 D2 .929

EXP =F3 = 1.000*F10 + .000 D3 1.000

IND_M =F4 = .894 F10 + .447 D4 .800

SOCI =F5 = .848*F10 + .530 D5 .719

G =F10 = .871*V3 + .491 D10 .759

COGN_SSS=F12 = .910*F20 + .415 D12 .828

COGN_S =F13 = .414*F20 + .910 D13 .171

HA_EV =F14 = .549 F20 + .836 D14 .302

COGN =F20 = .302*F10 + .122*D1 + .708*D2 + .088*D4

+ .144*D5 + .262 D20 + .233*D34 + .492*D35 .932

N =F21 = -.090*F30 + .996 D21 .008

C =F22 = .633*F30 + .774 D22 .400

O =F23 = .733 F30 + .680 D23 .538

A =F24 = .675*F30 + .738 D24 .455

E =F25 = .533*F30 + .846 D25 .284

GFP =F30 = .720*F20 + .694 D30 .518

T_EI_SK-=F31 = .668*F30 + .744 D31 .446

T_EI_STA=F32 = -.211*F30 + .977 D32 .045

T_EI_SEN=F33 = .572*F30 + .820 D33 .327

A_EI_REC=F34 = .899*F10 + .438 D34 .808

A_EI_DEF=F35 = .501*F10 + .866 D35 .251

GOODNESS OF FIT SUMMARY FOR METHOD = ML

MODEL AIC = -174.070 MODEL CAIC = -5789.731

CHI-SQUARE = 2327.930 BASED ON 1251 DEGREES OF FREEDOM

PROBABILITY VALUE FOR THE CHI-SQUARE STATISTIC IS .00000

COMPARATIVE FIT INDEX (CFI) = .997

ROOT MEAN-SQUARE ERROR OF APPROXIMATION (RMSEA) = .060

90% CONFIDENCE INTERVAL OF RMSEA ( .056, .063)

RELIABILITY COEFFICIENTS

------------------------

CRONBACH'S ALPHA = .805

**Model 3: Top-down mediation of cognizance between personality and cognitive factors.**

STANDARDIZED SOLUTION: R-SQUARED

SR1SELFR=V41 = .469 F13 + .883 E41 .220

SR2SELFR=V42 = .857*F13 + .516 E42 .734

SR50SELF=V90 = .453*F13 + .891 E90 .206

EI8 =V187= .636*F32 + .772 E187 .405

EI13 =V192= .668 F33 + .744 E192 .446

EI15 =V194= .734 F32 + .679 E194 .539

EI17 =V196= .609 F31 + .793 E196 .371

EI18 =V197= .622*F31 + .783 E197 .387

EI19 =V198= .636*F33 + .772 E198 .404

EI20 =V199= .627*F31 + .779 E199 .393

EI21 =V200= .516*F31 + .856 E200 .267

EI25 =V204= .782*F33 + .624 E204 .611

EI26 =V205= .609*F33 + .793 E205 .371

EI31 =V210= .572*F32 + .820 E210 .328

EI35 =V214= .511*F32 + .859 E214 .261

HAI_F1 =V279= .348 F14 + .938 E279 .121

HAI_SD1 =V280= .457*F14 + .889 E280 .209

HAI_MT5 =V282= .443*F14 + .896 E282 .196

HAI_S1 =V283= .258*F14 + .966 E283 .067

BOTTLE =V285= .715 F1 + .699 E285 .511

CAR =V286= .510*F1 + .860 E286 .260

NA =V288= .501 F2 + .866 E288 .251

NS =V289= .647*F2 + .762 E289 .419

SEEDS =V290= .638 F3 + .770 E290 .407

COMB =V291= .555*F3 + .832 E291 .308

MAT_1 =V292= .476 F4 + .879 E292 .227

MAT_2 =V293= .705*F4 + .709 E293 .497

SOC_A =V295= .805 F5 + .593 E295 .649

SOC_B =V296= .659*F5 + .752 E296 .434

MATH1 =V297= .363 F12 + .932 E297 .132

SUP1 =V299= .780*F12 + .626 E299 .608

SUP2 =V300= .710*F12 + .705 E300 .504

SOC2 =V302= .743*F12 + .669 E302 .552

SPOR =V305= .394*F12 + .919 E305 .155

F2ACHIE =V310= .743 F22 + .669 E310 .552

F2ORG =V311= .901*F22 + .434 E311 .811

F1EMO =V312= .484 F21 + .875 E312 .235

F1EGO =V313= .999*F21 + .033 E313 .999

F5PROS =V314= .578 F25 + .816 E314 .335

F5EXT =V316= .896*F25 + .445 E316 .802

F4HELP =V317= .681 F24 + .733 E317 .463

F4AGR =V318= .943*F24 + .333 E318 .889

F3INTE =V320= .999 F23 + .043 E320 .998

F3OPEN =V321= .480*F23 + .877 E321 .230

TCWL =V369= .355 F34 + .935 E369 .126

PEGD =V370= .573*F34 + .820 E370 .328

COMEM =V371= .769*F34 + .640 E371 .591

STORY =V372= .503*F34 + .864 E372 .253

FAC_JOY =V373= .418 F35 + .908 E373 .175

FAC_GRIF=V374= .526*F35 + .850 E374 .277

FAC_SURP=V375= .877*F35 + .480 E375 .769

SPAC =F1 = .534*F10 + .845 D1 .285

QUA =F2 = .967*F10 + .255 D2 .935

EXP =F3 = .994*F10 + .107 D3 .988

IND_M =F4 = .866 F10 + .499 D4 .751

SOCI =F5 = .844*F10 + .536 D5 .712

G =F10 = .932*F20 + .362 D10 .869

COGN_SSS=F12 = .412*F20 + .911 D12 .170

COGN_S =F13 = -.190*F20 + .982 D13 .036

HA_EV =F14 = .986 F20 + .169 D14 .971

COGN =F20 = -.407*F30 + .171 D20 + .172*D21 + .320*D22

+ .345*D23 + .434*D24 + .088*D25 + .284*D31

- .437*D32 + .294*D33 .971

N =F21 = -.091*F30 + .996 D21 .008

C =F22 = .636*F30 + .772 D22 .404

O =F23 = .633 F30 + .774 D23 .401

A =F24 = .535*F30 + .845 D24 .287

E =F25 = .489*F30 + .872 D25 .239

GFP =F30 = -.476*V3 + .880 D30 .226

T_EI_SK-=F31 = .567*F30 + .823 D31 .322

T_EI_STA=F32 = -.079*F30 + .997 D32 .006

T_EI_SEN=F33 = .493*F30 + .870 D33 .243

A_EI_REC=F34 = .906*F10 + .423 D34 .821

A_EI_DEF=F35 = .520*F10 + .854 D35 .270

GOODNESS OF FIT SUMMARY FOR METHOD = ML

MODEL AIC = 2.466 MODEL CAIC = -5617.684

CHI-SQUARE = 2506.466 BASED ON 1252 DEGREES OF FREEDOM

PROBABILITY VALUE FOR THE CHI-SQUARE STATISTIC IS .00000

COMPARATIVE FIT INDEX (CFI) = .996

ROOT MEAN-SQUARE ERROR OF APPROXIMATION (RMSEA) = .064

90% CONFIDENCE INTERVAL OF RMSEA ( .061, .068)

RELIABILITY COEFFICIENTS

------------------------

CRONBACH'S ALPHA = .805

**Model 4: Model testing effects on school performance.**

STANDARDIZED SOLUTION: R-SQUARED

SOCIAL =V342= .428*F11 + .904 E342 .183

TCWL =V369= .420 F34 + .908 E369 .176

PEGD =V370= .607*F34 + .795 E370 .369

COMEM =V371= .671*F34 + .741 E371 .450

STORY =V372= .396*F34 + .918 E372 .157

FAC_JOY =V373= .411 F35 + .912 E373 .169

FAC_GRIF=V374= .419*F35 + .908 E374 .175

FAC_SURP=V375= .877*F35 + .481 E375 .768

GREEK =V376= .964 F4 + .266 E376 .929

MATHS =V377= .855*F4 + .518 E377 .731

COGN =F1 = .222*F11 + .975 D1 .049

GFP =F2 = -.170*F11 + .587*D1 + .792 D2 .373

GEI =F3 = .441*F11 + .583*D1 + .452*D2 + .511 D3 .739

SCH =F4 = .170*F11 + .345*D1 + .350*D2 + .854 D4 .271

GF =F11 = .704*V3 + .710 D11 .496

COGN_SSS=F12 = .997 F1 + .075 D12 .994

COGN_S =F13 = .349*F1 + .937 D13 .122

HA_EV =F14 = .265*F1 + .752*D11 + .550 D14 .698

N =F21 = -.208*F211 + .978 D21 .043

C =F22 = .927*F211 + .374 D22 .860

O =F23 = .663*F212 + .749 D23 .439

A =F24 = .625 F211 + .781 D24 .390

E =F25 = .431 F212 + .903 D25 .185

T_EI_SK-=F31 = .730*F311 + .684 D31 .533

T_EI_STA=F32 = -.359*F311 + .933 D32 .129

T_EI_SEN=F33 = .719 F311 + .695 D33 .517

A_EI_REC=F34 = .677*F312 + .736 D34 .459

A_EI_DEF=F35 = .797 F312 + .604 D35 .635

A_STA =F211= .881*F2 + .473 D211 .776

B_PLA =F212= .989 F2 + .147 D212 .978

EIt =F311= .828*F3 + .560 D311 .686

EIA =F312= .995 F3 + .095 D312 .991

CORRELATIONS AMONG INDEPENDENT VARIABLES

---------------------------------------

E D

--- ---

E341- QUAL .329*I I

E282-SEA_MT5 I I

I I

GOODNESS OF FIT SUMMARY FOR METHOD = ML

MODEL AIC = -504.511 MODEL CAIC = -5026.663

CHI-SQUARE = 1701.489 BASED ON 1103 DEGREES OF FREEDOM

PROBABILITY VALUE FOR THE CHI-SQUARE STATISTIC IS .00000

COMPARATIVE FIT INDEX (CFI) = .994

ROOT MEAN-SQUARE ERROR OF APPROXIMATION (RMSEA) = .058

90% CONFIDENCE INTERVAL OF RMSEA ( .052, .063)

RELIABILITY COEFFICIENTS

------------------------

CRONBACH'S ALPHA = .802
